# *Cryptococcus neoformans* Glucuronoxylomannan and Sterylglucoside Are Required for Host Protection in an Animal Vaccination Model

**DOI:** 10.1128/mBio.02909-18

**Published:** 2019-04-02

**Authors:** Ana Caroline Colombo, Antonella Rella, Tyler Normile, Luna S. Joffe, Patricia M. Tavares, Glauber R. de S. Araújo, Susana Frases, Erika P. Orner, Amir M. Farnoud, Bettina C. Fries, Brian Sheridan, Leonardo Nimrichter, Marcio L. Rodrigues, Maurizio Del Poeta

**Affiliations:** a Instituto de Bioquímica Médica (IBqM), Rio de Janeiro, Universidade Federal do Rio de Janeiro, Rio de Janeiro, Brazil; b Instituto de Microbiologia Paulo de Góes (IMPG), Universidade Federal do Rio de Janeiro, Rio de Janeiro, Brazil; c Department of Molecular Genetics and Microbiology, Stony Brook University, Stony Brook, New York, USA; d Centro de Desenvolvimento Tecnológico em Saúde (CDTS), Fundação Oswaldo Cruz, Rio de Janeiro, Brazil; e Instituto de Biofísica Carlos Chagas Filho, Universidade Federal do Rio de Janeiro, Rio de Janeiro, Brazil; f Chemical and Biomolecular Engineering Department, Ohio University, Athens, Ohio, USA; g Department of Medicine, Division of Infectious Diseases, Stony Brook University, Stony Brook, New York, USA; h Instituto Carlos Chagas, Fundação Oswaldo Cruz (Fiocruz), Curitiba, Brazil; i Veterans Administration Medical Center, Northport, New York, USA; Washington University School of Medicine

**Keywords:** *Cryptococcus neoformans*, capsule, extracellular vesicles, fungal infection, glucuronoxylomannan, glycolipids, polysaccharides, sterylglucosides, vaccine, vesicles

## Abstract

Cryptococcus neoformans is an encapsulated fungal pathogen that causes meningoencephalitis. There are no prophylactic tools for cryptococcosis. Previously, our group showed that a C. neoformans mutant lacking the gene encoding sterylglucosidase (Δ*sgl1*) induced protection in both immunocompetent and immunocompromised murine models of cryptococcosis. Since sterylglucosidase catalyzes degradation of sterylglucosides (SGs), accumulation of this glycolipid could be responsible for protective immunity. In this study, we analyzed whether the activity of SGs is sufficient for the protective effect induced by the Δ*sgl1* strain. We observed that the accumulation of SGs impacted several properties of the main polysaccharide that composes the fungal capsule, glucuronoxylomannan (GXM). We therefore used genetic manipulation to delete the *SGL1* gene in the acapsular mutant Δ*cap59* to generate a double mutant (strain Δ*cap59*/Δ*sgl1*) that was shown to be nonpathogenic and cleared from the lung of mice within 7 days post-intranasal infection. The inflammatory immune response triggered by the Δ*cap59*/Δ*sgl1* mutant in the lung differed from the response seen with the other strains. The double mutant did not induce protection in a vaccination model, suggesting that SG-related protection requires the main capsular polysaccharide. Finally, GXM-containing extracellular vesicles (EVs) enriched in SGs delayed the acute lethality of Galleria mellonella against C. neoformans infection. These studies highlighted a key role for GXM and SGs in inducing protection against a secondary cryptococcal infection, and, since EVs notoriously contain GXM, these results suggest the potential use of Δ*sgl1* EVs as a vaccination strategy for cryptococcosis.

## Abstract (precis)

The number of deaths from cryptococcal meningitis is around 180,000 per year. The disease is the second leading cause of mortality among individuals with AIDS. Antifungal treatment is costly and associated with adverse effects and resistance, evidencing the urgency of development of both therapeutic and prophylactic tools. Here we demonstrate the key roles of polysaccharide- and glycolipid-containing structures in a vaccination model to prevent cryptococcosis.

## INTRODUCTION

Cryptococcosis is a fungal disease caused by the yeast-like pathogens Cryptococcus neoformans and C. gattii. The fungal cells initially infect the lungs, and disease progression results in a highly lethal form of meningoencephalitis. Cryptococcal meningitis is the second leading cause of mortality in AIDS patients, behind tuberculosis, with around 15% of deaths reported (1). Antifungal treatment of cryptococcosis can have many collateral effects and is sometimes associated with drug resistance (2, 3). There are no licensed prophylactic therapeutics to prevent cryptococcosis, although different vaccine strategies have been designed and tested in animal models (4–11).

Previous studies by our group have shown a potential vaccine effect in mice inoculated with a C. neoformans strain that contains disruption of the gene encoding the enzyme sterylglucosidase 1 (*SGL1*). This mutant strain accumulates sterylglucosides (SGs) (7). Protection was observed in CD4^+^ T cell-depleted mice, mimicking the conditions observed in AIDS patients (7). In fact, it has been shown that SGs play important roles in the stimulation of host immune responses (12, 13), such as an increase in cytokine production (14–16) and proliferation of lymphocytes and eosinophils (14, 17). The most abundant SG found in fungi is the ergosteryl-3-β-glucoside, but only small amounts are usually detectable (12, 18). Therefore, the Δ*sgl1* mutant represents a promising tool for immunomodulation studies due the accumulation of this lipid. It remains unknown whether SG accumulation is the only factor involved in the protection induced by the Δ*sgl1* mutant in a murine model of cryptococcosis.

Several fungal components can modulate the host immune system, inducing partial or complete protection (7, 10, 19–21). In *Cryptococcus* sp., glucuronoxylomannan (GXM), the most abundant polysaccharide composing the capsule, is a major immunomodulator (22, 23). Antibodies against GXM are ubiquitously found in the serum of adults and can also be detected in childhood (24–26). Although these antibodies are not necessarily associated with antibody-mediated protection, conjugation of GXM to tetanus toxoid (TT) and Pseudomonas aeruginosa recombinant exoprotein A (rEPA) can induce protective effects (11, 19, 27). GXM-based therapies have been developed to elicit protection or antifungal activity. For example, peptides that mimic GXM epitopes and antibodies specific for GXM can induce protection against C. neoformans (9, 28, 29).

The main objective of this study was to assess if GXM, in association with the accumulation of SGs, elicited protection in animals lethally infected with C. neoformans. Our results indicate that GXM plays an essential role in the SG-linked protection, with important implications in a vaccine formulation strategy.

## RESULTS

### SGs and GXM were functionally connected in C. neoformans.

**FIG 1:**
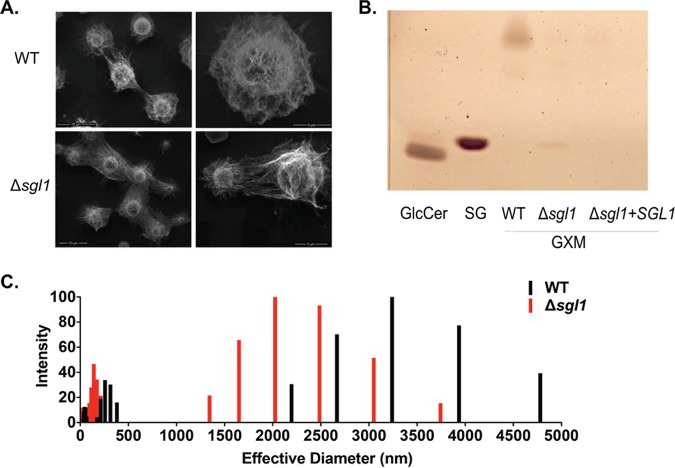
Accumulation of sterylglucosides (SGs) altered the structural and physical properties of glucuronoxylomannan (GXM). (A) Scanning electron microscopy (SEM) micrographs of WT C. neoformans and Δ*sgl1* cells cultivated in minimal medium for 48 h. Data show that Δ*sgl1* cells presented denser and thicker capsules than WT strain. (B) Thin layer chromatography (TLC) of purified exo-GXM of WT, Δ*sgl1*, and Δ*sgl1+SGL1* reconstituted strains. Glucosylceramide (GlcCer) and SG extracts were used as controls. A band was identified for mutant *Δsgl1*, indicating the presence of SGs in the purified exo-GXM. (C) Dynamic light scattering (DLS) shows that the purified exo-GXM fibers of mutant Δ*sgl1* were smaller than those of the WT strain.

We studied the effect of SG accumulation on the capsule structure and GXM physical properties. We compared morphological features of the wild-type (WT) and Δ*sgl1* strains by scanning electron microscopy (SEM). The capsule of the Δ*sgl1* strain was thicker and more branched, connecting different cells (Fig. 1A). The suggestion that SG accumulation affected capsular architecture led us to evaluate the association between these glycolipids and GXM. Therefore, we isolated GXM from culture supernatants (exo-GXM) and searched for the presence of SGs in polysaccharide fractions by mass spectrometry (MS) and thin-layer chromatography (TLC). Since GXM is exported in EVs, these membranous compartments were included in this analysis. Cell pellets were included as controls. In samples obtained from the Δ*sgl1* mutant, SGs were approximately 45-fold, 1,000-fold, and 2,500-fold more abundant in cell pellets, exo-GXM, and EVs, respectively, than in similar fractions obtained from WT cells (Table 1). MS results with GXM samples were confirmed by TLC (Fig. 1B). The dimensions of GXM obtained from WT and mutant cells were analyzed by dynamic light scattering (DLS), which revealed that Δ*sgl1* exo-GXM fibers were smaller than similar fractions obtained from WT cells (Fig. 1C). The supernatant of strain Δ*sgl1* from the GXM purification protocol showed an obvious increase in viscosity compared to the Δ*sgl1+SGL1* reconstituted strain (see Video S1 in the supplemental material). Since GXM composition and extracellular concentration were not altered in mutant cells (see Fig. S1 in the supplemental material), we attributed the differences observed here to the accumulation of SGs.

**TABLE 1:** Quantitative analysis by mass spectrometry of intracellular and secreted SGs of two experiments

Strain	Ergosteryl-3 β-glucoside (pmol/Pi)					
	Cells (*V*_i_ = 10 ml)^*a*^		GXM (*V*_i_ = 1 ml)		EVs (*V*_i_ = 50 µl)	
WT		3.96	0.004	0.013	0.0008	-
Δ*sgl1*	249.92	121.26	10.28	10.45	2.47	1.87

a *V*_i_, initial volume.

10.1128/mBio.02909-18.2 FIG S1 GXM secretion and composition analysis. (A) Culture supernatant was aliquoted after 24 h or 48 h of cultivation of fungal cells. Enzyme-linked immunosorbent assay (ELISA) results revealed no difference between the WT and Δ*sgl1* strains under different temperature conditions. (B) Purified exo-GXM was analyzed by gas chromatography-mass spectrometry (GC-MS). No significant differences were observed among the WT, Δ*sgl1*, and Δ*sgl1+SGL1* reconstituted strains. Data for GXM secretion are represented by means ± standard deviations (SD). Download FIG S1, PDF file, 0.04 MB. Copyright © 2019 Colombo et al. 2019 Colombo et al. This content is distributed under the terms of the Creative Commons Attribution 4.0 International license.

10.1128/mBio.02909-18.7 MOVIE S1 Viscosity of strain Δ*sgl1* and strain Δ*sgl1+SGL1* supernatants from GXM purification protocol. Download Movie S1, MOV file, 19.4 MB. Copyright © 2019 Colombo et al. 2019 Colombo et al. This content is distributed under the terms of the Creative Commons Attribution 4.0 International license.

### Deletion of *SGL1* in an acapsular strain (Δ*cap59*) resulted in SG accumulation without affecting cellular morphology.

**FIG 2:**
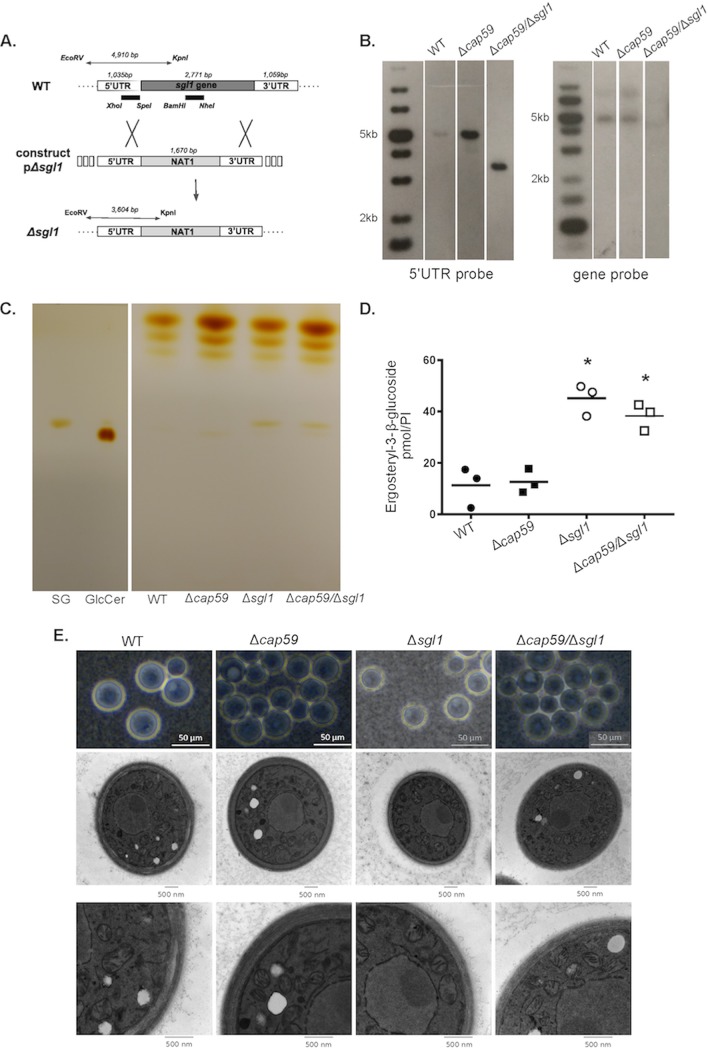
Deletion of *SGL1* gene in acapsular strain Δ*cap59* and phenotypic analysis. (A) Strategy for the deletion of *SGL1* gene using a nourseothricin (NAT1) cassette. Restriction enzymes and the size of the DNA fragment are represented. (B) Southern blot analysis of genomic DNA using the 5′UTR and a gene probe, which are represented by a black bar in panel A. Transformant 18 was named mutant Δ*cap59*/Δ*sgl1*. The double mutant showed an expected band of 3,604 bp and no apparent band when 5′UTR and gene probes were used, respectively. (C) TLC of lipids extracted from Δ*cap59*/Δ*sgl1*, WT, Δ*cap59*, and Δ*sgl1* cells was performed. The presence of bands representing the size of SGs was observed for both *SGL1*-deleted mutants, indicating the accumulation of SGs in these mutants. (D) MS analysis of lipids extracted from the fungal cells performed in triplicate showed a 3-fold increase in the levels of SGs in the *SGL1*-deleted mutants compared to the WT and Δ*cap59* strains, confirming the TLC result. *, *P* < 0.003 (compared to the WT and Δ*cap59* strains). (E) India ink and transmission electron microscopy (TEM) analysis of the fungal cells grown in YPD for 24 h. Absence of capsule was confirmed for the double mutant and strain Δ*cap59*. Acapsular cells presented with formation of clumps. GlcCer, glucosylceramide.

The presumed association between SGs and extracellular GXM led us to delete *SGL1* in a mutant lacking a putative regulator of capsular export (*CAP59*) (30) for further functional analyses. After biolistic DNA delivery, we selected 31 of 280 colonies that were nourseothricin (NAT) resistant and confirmed the correct plasmidial insertion and disruption of the *SGL1* gene via Southern blot analysis. Transformant 18 showed the expected band of 3,604 bp (Fig. 2A and B). To confirm the phenotype that had been associated with the Δ*sgl1* mutant, we performed TLC and MS analyses (Fig. 2C and D). The single and double mutants manifested similar levels of SG accumulation, and similar amounts of SGs were observed in the WT and Δ*cap59* strains. The amounts of SGs seen in mutants lacking the *SGL1* gene were increased approximately 3-fold in comparison to WT and Δ*cap59* cells (*P* < 0.005). No significant changes were observed in sphingolipid glucosylceramide and inositol phosphorylceramide (IPC) detection, indicating that the mutant cells lacking *SGL1* did not manifest general defects in sphingolipid biosynthesis (data not shown). Morphological analyses by microscopy confirmed a lack of capsular structures in the mutants lacking *CAP59* (Fig. 2E). Noticeably, these cells also formed large clumps. On the basis of its genetic and morphological properties, transformant 18 was selected for further analyses and named the Δ*cap59*/Δ*sgl1* strain.

**FIG 3:**
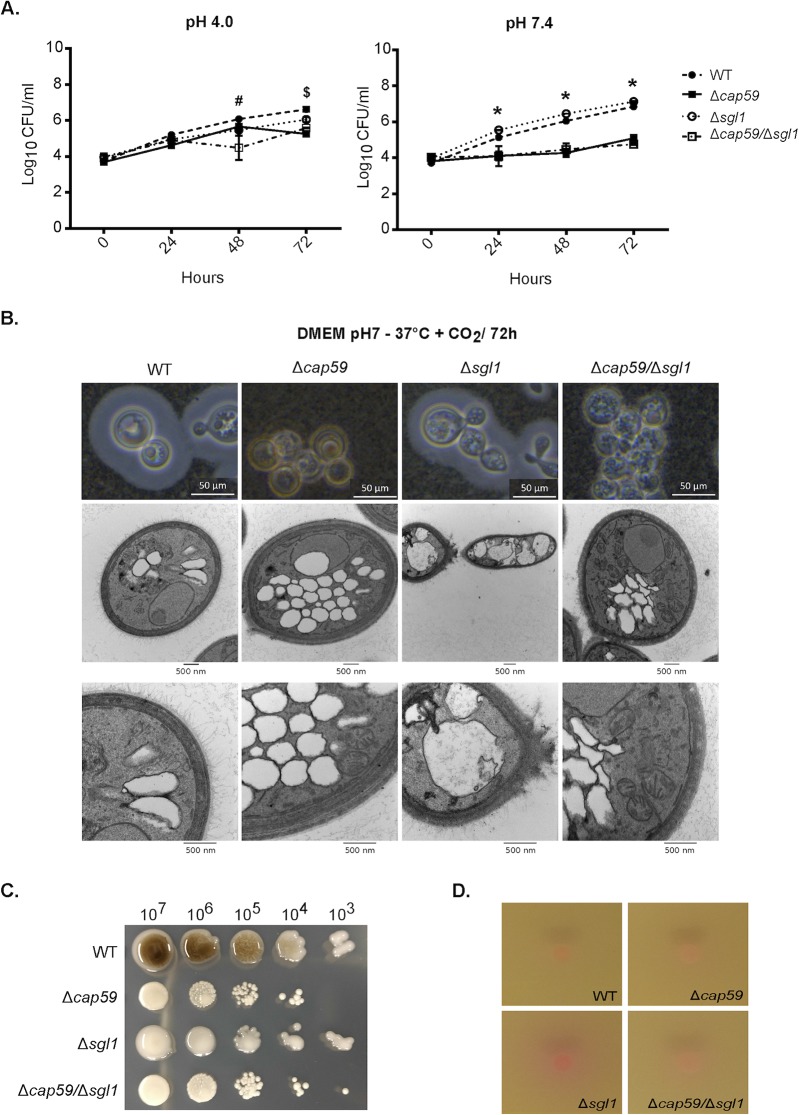
The double mutant (Δ*cap59*/Δ*sgl1*) displayed characteristics similar to those displayed by the corresponding Δ*cap59* background strain under host-mimicking conditions. (A) Growth curve of the fungal cells cultivated in DMEM plus 5% CO_2_ at 37°C and pH 4.0 (lysosomal milieu; left panel) or pH 7.4 (extracellular milieu; right panel). An impairment of growth was observed by comparisons between the acapsular and capsular strains only at pH 7.4. Data represent means ± standard deviations (SD) of results from an experiment performed in triplicate. #, difference between *Δcap59/Δsgl1* strain compared to the other strains (*P* < 0.001). $, difference between the WT strain and the acapsular mutants (*P* < 0.001). *, difference between the capsular and acapsular mutants (*P* < 0.001). (B) India ink and TEM were performed at pH 7.0 since growth impairment was observed. Data show that the Δ*cap59*/Δ*sgl1* cells did not show any evident changes. However, the Δ*cap59* strain displayed a higher number of vacuoles and the Δ*sgl1* strain some cells with elongated shape and big vacuoles with fibrillar-like structures inside. (C) The melanin production activity of all the mutants was impacted compared to the WT strain results. (D) Christensen's urea agar was used for urea formation analysis. The strain Δ*cap59*/Δ*sgl1* results showed similarity to the Δ*cap59* mutant results.

Proliferation analyses demonstrated that the Δ*cap59*/Δ*sgl1* and Δ*cap59* strains manifested similar decreased growth rates in DMEM in a 5% CO_2_ atmosphere (37°C), particularly at pH 7.4. India ink counterstaining under these conditions revealed Δ*cap59*/Δ*sgl1* cells with clumped morphology, like strain Δ*cap59* (Fig. 3A). The analyses performed at pH 7.4 showed the most pronounced growth defects, and further transmission electron microscopy (TEM) analysis revealed that Δ*cap59* cells had greater numbers of vacuoles than all the other strains (Fig. 3B). The Δ*sgl1* mutant had both elongated shaping and bigger vacuoles with internal fibrillar-like structures. Additional phenotypic characterization included melanin and urease analyses. At 37°C and 5% CO_2_, all the mutants displayed impaired melanin production compared to WT cells (Fig. 3C). Urease assays revealed similarity among the Δ*cap59*/Δ*sgl1*, Δ*cap59*, and WT strains and a slight increase in enzyme activity in the Δ*sgl1* mutant (Fig. 3D).

### The Δ*cap59*/Δ*sgl1* mutant was nonpathogenic and was cleared from the lungs 7 days after intranasal injection in a mouse model.

**FIG 4:**
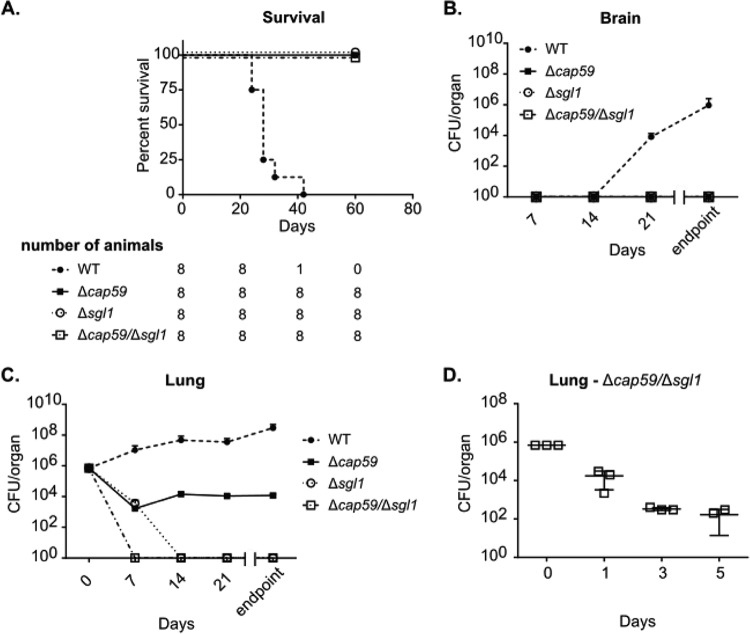
The Δ*cap59*/Δ*sgl1* mutant was nonpathogenic and was cleared from the lung after intranasal infection in a mouse model. (A) Survival curve of CBA/J mice intranasally inoculated with 7 × 10^5^ C. neoformans cells. The mutants were nonpathogenic, whereas the WT strain caused the animals to succumb to the infection (*n* = 8 per treatment). (B) Fungal burden was assessed by CFU counting of homogenized brains. Only WT cells were able to reach the brain. Also of note, the CFU for the WT strain was verified on different days (days 7, 11, 17, and 28) due to the higher susceptibility to inoculation (*n* = 3 per group in each time point). (C) The CFU counting of the lungs was performed under the same conditions as described for the brain. The Δ*cap59*/Δ*sgl1* and Δ*sgl1* cells disappeared from the lungs on days 7 and 14 postinfection, respectively. The Δ*cap59* cells decreased in number but were maintained in the lungs, whereas the WT cell numbers increased exponentially. (D) Fungal burden of lungs inoculated with the double mutant to assess early time points (since this mutant was cleared from the lungs by day 7 postinfection).

Previous studies have shown that both the Δ*cap59* and Δ*sgl1* mutants were nonpathogenic in intranasal infection models in mice (7, 31). We reproduced these models with the double mutant in the present study. CBA/J mice were susceptible to infection with WT cells, but all animals survived after intranasal inoculation of the mutant strains (Fig. 4A). Fungal loads (CFU counting) were determined 7, 14, 21, and 60 days postinoculation with the mutant strains and 7, 11, 17, and 28 days postinoculation with the WT strain. Only the WT cells were able to reach the brains of the animals (Fig. 4B). Although all mutants were nonpathogenic and unable to reach the brain, the lung fungal burdens differed considerably (Fig. 4C). While CFU levels increased exponentially in mice infected with the WT strain, the Δ*cap59*/Δ*sgl1* double mutant was cleared from the lungs 7 days after inoculation. The Δ*cap59* burden was decreased 7 days after inoculation but remained detectable in lung samples throughout the observation. The Δ*sgl1* mutant was also cleared from the lungs, but only 14 days postinfection. To verify if the double mutant was eliminated from the lungs before day 7 postinfection, we also analyzed CFU levels on days 1, 3, and 5 postinoculation with the Δ*cap59*/Δ*sgl1* strain. A time-dependent decrease of CFU levels was observed (Fig. 4D).

### Acapsular cells were mainly found inside bronchioles and induced a similar immune response when compared to capsular cells.

**FIG 5:**
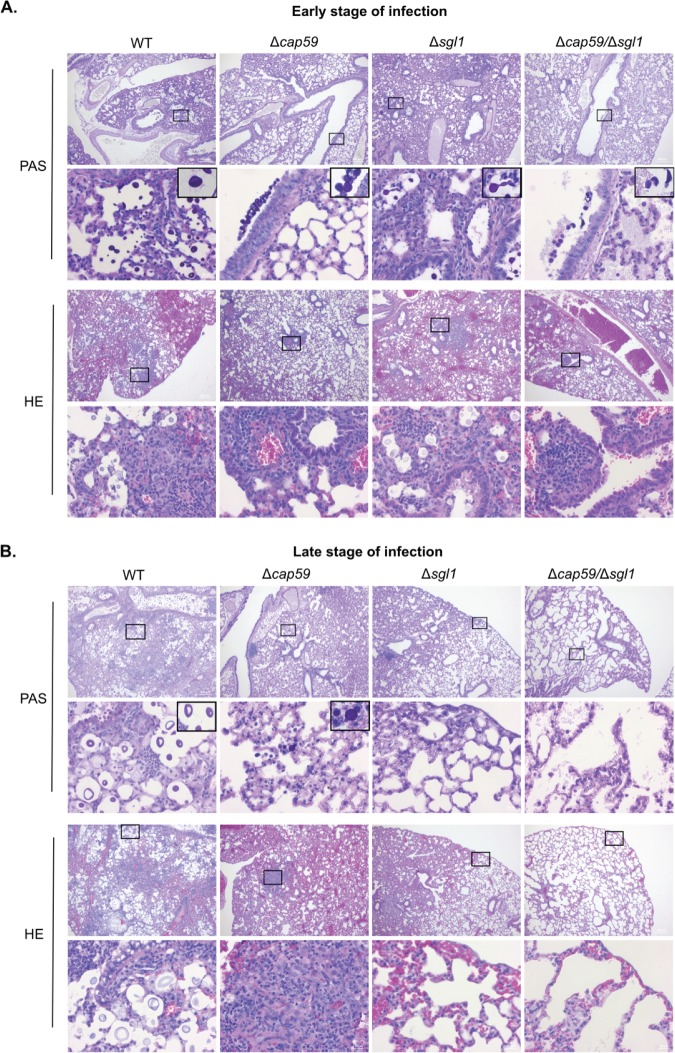
Lung histology in early and late stages of infection. CBA/J mice were inoculated intranasally with 7 × 10^5^ C. neoformans cells. After 3 days (A) or 14 days (for the WT strain) and 60 days (for the mutants) (B), lungs were removed, fixed, and paraffin embedded. Periodic acid–Schiff (PAS) and hematoxylin and eosin (HE) staining was performed. Pictures were taken using magnifications of ×5 and ×40. Fungal cells observed at a magnification of ×100 are highlighted in the upper right corner of selected panels. Early during the infection, the different inoculum treatments resulted in fungal cells in intimate contact with immune cell infiltrates, whereas later during the infection, the *SGL1-*deleted mutant cells were not found and lower numbers of immune cells and emphysema were observed.

**FIG 6:**
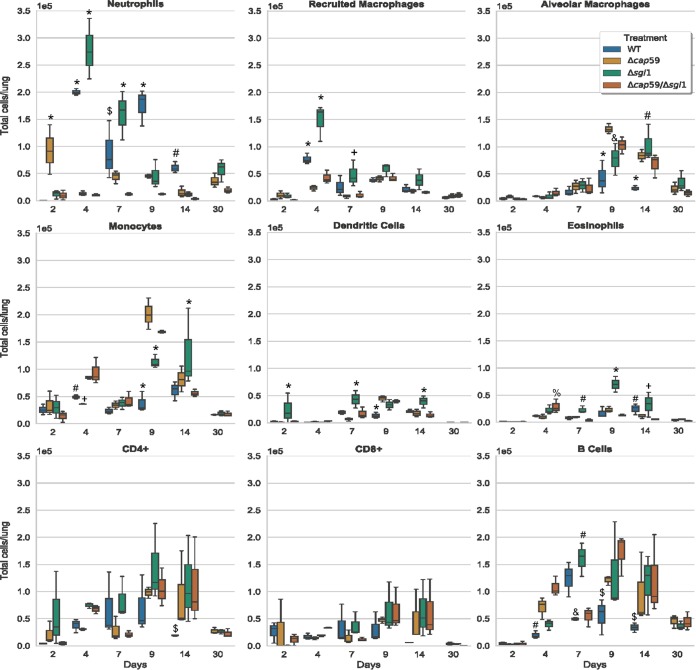
Lung immune cell counting during the course of infection. CBA/J mice were inoculated intranasally with 5 × 10^5^ C. neoformans cells. Lungs were removed and processed for flow cytometry analysis on days 2, 4, 7, 9, 14, and 30 postinfection. At the early stages, there was an increase in the numbers of neutrophils and recruited macrophages on day 4 postinfection in mice infected with capsular mutants. Although the Δ*cap59* mutant induced an increase of neutrophil numbers at day 2, a marked decrease was observed afterwards. The Δ*sgl1* mutant induced an even higher level of cell infiltration than did the WT at day 4. This mutant also triggered higher numbers of monocytes and eosinophils (at 9 and 14 days) and dendritic cells (at 2, 7, and 14 days) than were seen with the other groups. At 4 days postinfection, the Δ*cap59*/Δ*sgl1* mutant induced an increase in the levels of monocytes and eosinophils compared to the WT and Δ*cap59* strains. At later stages of infection, no clear difference was observed among the mutants. However, all the mutants, including the *SGL1-*deleted mutants, induced an increase in the number of lymphocytes and alveolar macrophages compared to WT-infected mice, respectively (9 and/or 14 days). Furthermore, the double mutant triggered an immune response similar to that triggered by the Δ*cap59* strain. At the same time point, *P* < 0.05 (asterisks [*] indicate results different from those seen with the other treatments; number signs [#] indicate results different from those seen with the Δ*cap59*/Δ*sgl1* mutant; dollar signs [$] indicate results different from those seen with the other strains except for the Δ*cap59* mutant; plus signs [+] indicate results different from those seen with the other strains except for the WT strain; ampersands [&] indicate results different from those seen with the other strains except for the Δ*cap59*/Δ*sgl1* mutant; percent signs [%] indicate results different from those seen with the other strains except for the Δ*sgl1* mutant). (*n* = 3 animals/treatment for each time point except for day 30, when WT-injected mice were found to have previously succumbed to the infection.)

**FIG 7:**
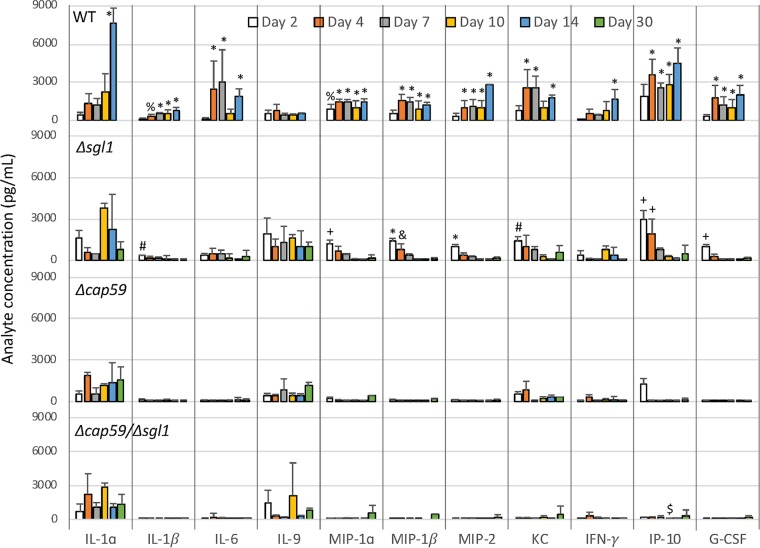
Lung cytokine levels during the course of infection. CBA/J mice were inoculated intranasally with 5 × 10^5^ C. neoformans cells. On days 2, 4, 7, 10, 14, and 30 postinfection, bronchoalveolar lavage fluid was first collected and then the lungs were removed and processed for cytokine quantification. Increases in the levels of IL-1β, G-CSF, CXCL10 (IP10), CCL3 (MIP1-α), CCL4 (MIP1-β), CXCL1 (KC), and CXCL2 (MIP2) were observed early in the infection after Δ*sgl1* treatment, and a subsequent, day-by-day return to basal levels was observed. Although WT-infected mice showed increased levels of these cytokines at early stages, high levels were maintained during the course of the infection. High levels of IL-6 (at 4, 7, and 14 days) and of IFN-γ and IL-1α (at 14 days) were also recorded for the WT-infected group. At the same time point, *P* < 0.05 (asterisks [*] indicate results different from those seen with the other treatments; number signs [#] indicate results different from those seen with the Δ*cap59*/Δ*sgl1* mutant; dollar signs [$] indicate results different from those seen with the other strains except for the Δ*cap59* mutant; plus signs [+] indicate results different from those seen with the other strains except for the WT strain; ampersands [&] indicate results different from those seen with the other strains except for the Δ*cap59*/Δ*sgl1* mutant; percent signs [%] indicate results different from those seen with the other strains except for the Δ*sgl1* mutant). (*n* = 3 animals/treatment for each time point except for day 30, when WT-infected mice had previously succumbed to the infection.)

Histologic analysis of the lungs at the beginning of infection (day 3) and at the endpoint (day 14 for the WT and day 60 for the mutants) revealed that all strains were detectable in the lungs in early infection points (Fig. 5A). However, the acapsular mutants, i.e., strains Δ*cap59*/Δ*sgl1* and Δ*cap59*, were mostly found inside the bronchioles. WT and Δ*sgl1* strains colonized the lung tissue, and big clusters of immune cells were observed. The presence of neutrophils and macrophages indicated the initiation of a pronounced inflammatory response. We confirmed the infiltration of the neutrophils and recruited macrophages by flow cytometry after the infection with the capsular fungal cells (Fig. 6). The Δ*sgl1* mutant induced higher number of monocytes and eosinophils (9 and 14 days) and dendritic cells (2, 7 and 14 days) than were seen with the other groups. At 4 days postinfection, the Δ*cap59*/Δ*sgl1* mutant induced an increase in monocytes and eosinophils compared to the WT and Δ*cap59* strains. Moreover, the double mutant triggered an immune response similar to that seen with the Δ*cap59* strain. In the lungs, the levels of proinflammatory cytokines, including interleukin-1β (IL-1β) and granulocyte colony-stimulating factor (G-CSF), and of chemokines, including IP10/CXCL10, MIP1-α/CCL3, MIP1-β/CCL4, MIP2/CXCL2, and KC/CXCL1, were increased and remained high after the infection with WT cells (Fig. 7). Interestingly, the levels of the same molecules were increased at early points after Δ*sgl1* infection; however, they subsequently decreased over the duration of the infection. These results indicate that sustaining high levels of these cytokines may stimulate a hyperinflammatory reaction, which could be associated with nonresolution of the infection and, ultimately, with mouse death. Although the host was able to mount an immunological response to the double mutant, the response was insufficient to induce protection, which was found to be more similar to the level induced by the acapsular Δ*cap59* mutant.

At the endpoints, however, only the WT and Δ*cap59* cells were found in infected tissues, which supports the CFU data (Fig. 5B). The lungs of animals infected with Δ*cap59* cells were affected only partially, while the damage associated with the WT infection was more widespread in the lungs. Infections with the Δ*cap59*/Δ*sgl1* mutant resulted in enlargement and partial destruction of the alveolar space, despite the fact that no fungal cells were found to be present there. Although we did not observe a clear difference in cell infiltration responses in the lungs of mice infected with the various mutants at the late stage of infection, we did see that the infections with the *SGL1-*deleted mutants induced an increase in the number of lymphocytes and alveolar macrophages compared to the results seen with the WT-infected mouse (9 and/or 14 days). There were noticeable differences in the cytokine levels between the mice infected with the WT strain and those infected with the mutant strains. In addition to corroborating the CFU data, these histological and immunological findings suggest that the lung immune response to the Δ*cap59*/Δ*sgl1* mutant was different from the immune response to the Δ*sgl1* mutant. The Δ*sgl1* mutant-induced cytokine and chemokine responses led to recruitment of leukocytes, mainly neutrophils and macrophages, with a subsequent decrease in the levels of inflammatory stimuli over the course of the infection, which may be the basis of the process by which this mutant is eliminated from the lung compared to WT.

In addition to the lung homogenate, we also analyzed levels of cytokine production from blood serum (Fig. S2A) and bronchoalveolar lavage (BAL) fluid (Fig. S3A) for the different infections. The production of cytokines in the BAL fluid was equivalent to the lung tissue results. However, we did not observe any explicit differences in the cytokine profiles in the serum of WT and Δ*sgl1* mutant-treated mice as seen in the lung homogenate, although we did see greater immune response stimulation in animals infected with the WT strain than in those infected with the mutants. The quantitative assessment of immune cells for the blood revealed an increase in the levels of lymphocytes of Δ*cap59*/Δ*sgl1* mutant-injected mice after infection resolution (day 10) (Fig. S4A). Collectively, these data suggest that the double mutant was able to induce an immune response, although to a lesser degree than the Δ*sgl1* mutant.

10.1128/mBio.02909-18.3 FIG S2 Blood cytokine levels during the course of infection and after WT challenge in the vaccination model. (A) During the infection, we did not observe any particular cytokine profile differences between the WT and Δ*sgl1* strains as observed in the lung homogenate; however, we did see a greater immune response assembled in animals infected with the WT strain compared to the other groups. (*n* = 3 animals/treatment for each time point, except for day 30, when WT-injected mice were found to have previously succumbed to the infection). (B) In the vaccination model, there were noticeable differences, mainly at late stages, in the cytokine profiles of the mice that were pretreated with the Δ*sgl1* strain versus the other groups. (*n* = 3 animals/treatment for each time point, except for day 30, when only Δ*sgl1* mutant-vaccinated mice were found to have survived after the WT challenge.) Download FIG S2, PDF file, 0.07 MB. Copyright © 2019 Colombo et al. 2019 Colombo et al. This content is distributed under the terms of the Creative Commons Attribution 4.0 International license.

10.1128/mBio.02909-18.4 FIG S3 BAL fluid cytokine levels during the course of infection and after WT challenge in the vaccination model. (A) During the infection, the production profile of cytokines in the BAL fluid was comparable to that seen in the lung tissue. (*n* = 3 animals/treatment for each time point, except for day 30, when WT-injected mice were found to have previously succumbed to the infection). (B) In the vaccination model, we did observe a distinct cytokine profile among the groups and an increase of cytokine levels at late stages only in PBS-pretreated animals, although only Δ*sgl1* mutant-vaccinated mice survived. (*n* = 3 animals/treatment for each time point, except for day 30, when only Δ*sgl1* mutant-vaccinated mice were found to have survived after the WT challenge.) Download FIG S3, PDF file, 0.07 MB. Copyright © 2019 Colombo et al. 2019 Colombo et al. This content is distributed under the terms of the Creative Commons Attribution 4.0 International license.

10.1128/mBio.02909-18.5 FIG S4 Blood immune cell counting during the course of infection and after WT challenge in the vaccination model. (A) We did observe an increase in the number of lymphocytes in the blood of Δ*cap59*/Δ*sgl1* mutant-injected mice after infection resolution (day 10) (*n* = 3 animals/treatment for each time point, except for day 30, when WT-injected mice were found to have previously succumbed to the infection). (B) In the vaccination model, we did see a decrease in the lymphocyte cell populations in Δ*sgl1* mutant-pretreated mice compared to the other pretreated animals. (*n* = 3 animals/treatment for each time point, except for day 30, when only Δ*sgl1* mutant-vaccinated mice were found to have survived after the WT challenge.) Download FIG S4, PDF file, 0.1 MB. Copyright © 2019 Colombo et al. 2019 Colombo et al. This content is distributed under the terms of the Creative Commons Attribution 4.0 International license.

### GXM is required for SG-related protection in a vaccination murine model.

**FIG 8:**
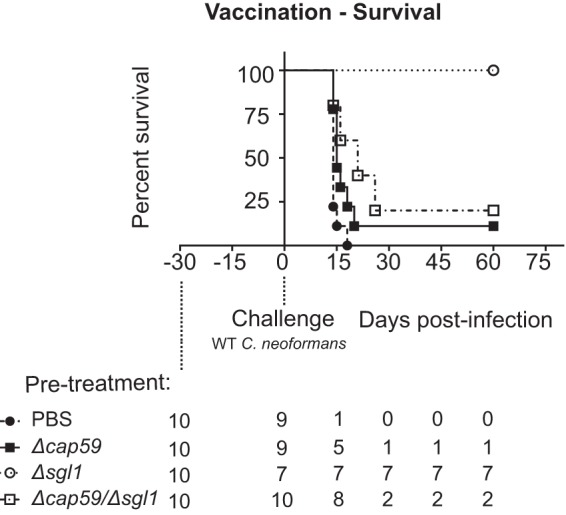
GXM is required for protection in a vaccination murine model involving SG accumulation. CBA/J mice were inoculated intranasally with 20 µl of PBS or 7 × 10^5^ cells of the Δ*cap59*, Δ*sgl1*, or Δ*cap59*/Δ*sgl1* strain. After 30 days, animals received an intranasal challenge of 7 × 10^5^ WT cells, and host survival was monitored for 60 days. The Δ*sgl1* mutant resulted in 100% protection of the animals (*, *P* < 0.0001). Vaccination with the Δ*cap59*/Δ*sgl1* double mutant culminated in 80% mortality (*, *P* = 0.0014 [versus PBS] or *P* = 0.0021 [versus strain *Δsgl1*]). PBS-treated animals succumbed to the infection after WT challenge. Only 1 animal pretreated with the Δ*cap59* mutant survived. No differences were observed compared to Δ*cap59*/Δ*sgl1* mutant (*P* = 0.1504). (*n* = 10 animals per treatment; some animals died due to anesthesia administered for the WT challenge.)

**FIG 9:**
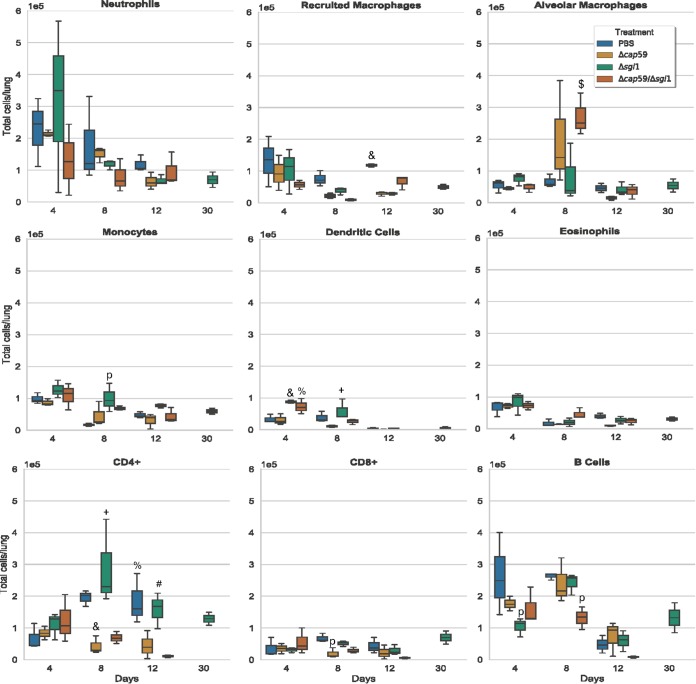
Lung immune cell counting after WT challenge in the vaccination model. CBA/J mice were pretreated intranasally with PBS or 5 × 10^5^ C. neoformans mutant cells. After 30 days, animals were challenged with the WT strain. On days 4, 8, 12, and 30 postinfection, lungs were removed and processed for flow cytometry analysis. There were observed increases in the dendritic cell (DC) and CD4^+^ T cell populations (day 8) in mice vaccinated with the Δ*sgl1* mutant but not in those vaccinated with the Δ*cap59* mutant or the double mutant (Δ*cap59*/Δ*sgl1*). At the same time point, *P* < 0.05 (plus signs [+] indicate results different from those seen under the other conditions except for the PBS treatment; number signs [#] indicate results different from those seen with the Δ*cap59*/Δ*sgl1* mutant; ρ indicate results different from those seen with PBS; dollar signs [$] indicate results different from those seen with the other strains except for the Δ*cap59* mutant; ampersands [&] indicate results different from those seen with the other strains except for the Δ*cap59*/Δ*sgl1* mutant; percent signs [%] indicate results different from those seen with the other strains except for the Δ*sgl1* mutant). (*n* = 3 animals/treatment for each time point except for day 30, when only Δ*sgl1* mutant-vacciated mice had survived after the WT challenge.)

**FIG 10:**
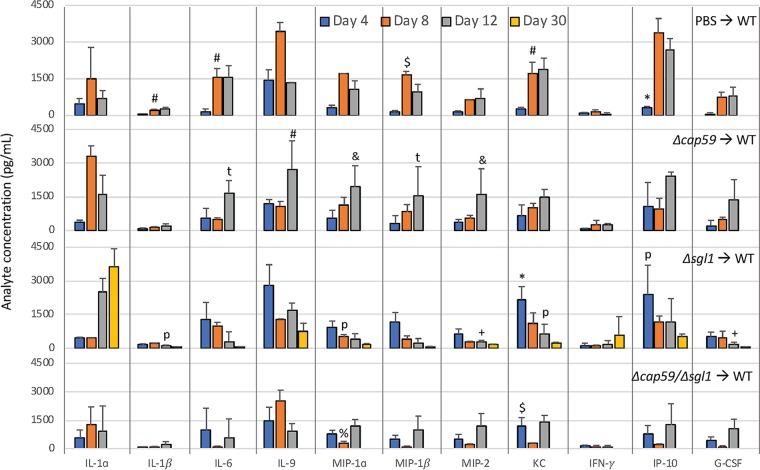
Lung cytokine levels after WT challenge in the vaccination model. CBA/J mice were pretreated intranasally with PBS or 5 × 10^5^ C. neoformans mutant cells. After 30 days, animals were challenged with the WT strain. On days 4, 8, 12, and 30 postinfection, bronchoalveolar lavage fluid was first collected and then lungs were removed and processed for cytokine quantification. High levels of IP10 at day 4 represented the only indication of any pretreatment condition (vaccination). An increase of CXCL1 (KC) levels and a tendency of increases in the levels of IL-6, CCL3 (MIP1-α), CCL4 (MIP1-β), and CXCL2 (MIP2) were observed in the Δ*sgl1* mutant-treated lungs compared to the other groups, and the mice infected with the Δ*sgl1* strain showed a tendency toward a decrease in the levels of these cytokines at each subsequent time point. At the same time point, *P* < 0.05 (asterisks [*] indicate results different from those seen with the other treatments; number signs [#] indicate results different from those seen with the Δ*cap59*/Δ*sgl1* mutant; ρ indicates results different from those seen with PBS; τ indicates results different from those seen with the Δ*sgl1* mutant; dollar signs [$] indicate results different from those seen with the other strains except for the Δ*cap59* mutant; plus signs [+] indicate results different from those seen under the other conditions except for the PBS treatment; ampersands [&] indicate results different from those seen with the other strains except for the Δ*cap59*/Δ*sgl1* mutant; percent signs [%] indicate results different from those seen with the other strains except for the Δ*sgl1* mutant). (*n* = 3 animals/treatment for each time point except for day 30, when only Δ*sgl1* mutant-vaccinated mice were found to have survived after the WT challenge.)

Our group has shown that exposure of mice to the Δ*sgl1* mutant triggers a protective immune response after challenge with a WT strain in both normal and immunocompromised (CD4^+^ cell-depleted) mice (7). GXM is known to induce antibody-mediated immunity against C. neoformans (11, 19, 27). Due to the connections between SGs and GXM described in this study, we tested the Δ*cap59*/Δ*sgl1* mutant in a vaccination model consisting of intranasal infection of CBA/J animals with the live attenuated Δ*cap59*/Δ*sgl1* strain followed by a rechallenge with the WT strain 30 days later and assessed survival for an additional 60 days (Fig. 8). As previously shown, the Δ*sgl1* mutant induced 100% protection in animals (*, *P* < 0.0001). However, vaccination with Δ*cap59*/Δ*sgl1* double mutant resulted in significantly (80%) higher mortality (*, *P* = 0.0014 [versus phosphate-buffered saline {PBS}]; *, P = 0.0021 [versus *Δsgl1*]). As expected, PBS-treated animals succumbed to the infection after rechallenge with WT cells. Only one animal survived vaccination with the Δ*cap59* mutant, which was similar to the results seen with animals immunized with the Δ*cap59*/Δ*sgl1* strain (*P* = 0.1504). To get a better understanding of the induced protection, we examined the immunological response through flow cytometry (Fig. 9) and cytokine analysis (Fig. 10) after the rechallenge with WT in the vaccination model. We did observe an increase in the levels of dendritic cells and CD4^+^ T cells (day 8) in mice vaccinated with the Δ*sgl1* strain but not in those vaccinated with the Δ*cap59* and the Δ*cap59*/Δ*sgl1* strains. We also observed the same profile of increased cytokine levels during the early stages of infection, which returned to basal levels as the infection progressed for Δ*sgl1* mutant-treated lungs compared to the other groups. These cytokines included CXCL1 (KC), IL-6, CCL3 (MIP1-α), CCL4 (MIP1-β), and CXCL2 (MIP2). We saw not only a lower number of lymphocyte cells in the blood samples of Δ*sgl1* mutant-pretreated mice (Fig. S4B) but also noticeable differences in the cytokine profiles, mainly at late stages, of mice that were pretreated with this strain versus the other groups (Fig. S2B). In the BAL fluid analysis, however, we did not find any similarity in the levels of cytokines to those determined in the lung homogenate analysis. We saw an increase of cytokine levels only at late stages in PBS-pretreated animals, although only Δ*sgl1* mutant-vaccinated mice survived (Fig. S3B). Altogether, these results indicate that GXM is required for protection in mice immunized under conditions of SG accumulation.

### Pretreatment with EVs enriched in SGs and GXM delayed acute lethality in an invertebrate model of infection.

**FIG 11:**
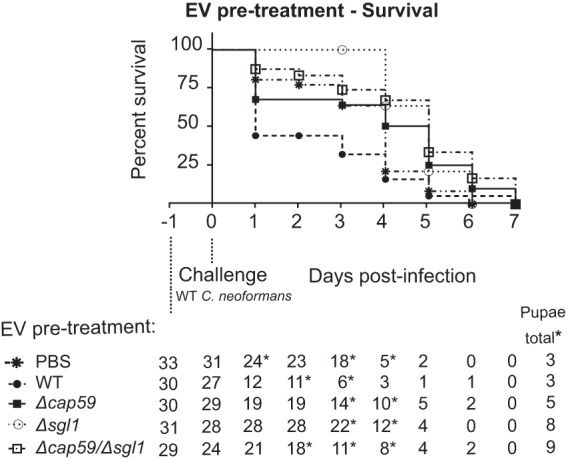
Extracellular vesicle (EV) pretreatment in an invertebrate model of C. neoformans infection. Around thirty Galleria mellonella larvae were injected through the proleg with 10 μl of PBS or an EV suspension of the WT, Δ*cap59*, Δ*sgl1*, or Δ*cap59*/Δ*sgl1* strain containing 2 μM sterol. After 24 h, animals received an injection of 2 × 10^4^ WT cells, and host survival was monitored. G. mellonella that had changed to pupae during the experiment were eliminated from the analysis. Animals that received WT EVs died sooner than the ones that received PBS treatment only (*, *P* = 0.0062). Although all animals died in the end of the experiment, Δ*sgl1* mutant EV pretreatment guaranteed 100% protection compared to PBS until day 3 (*, *P* = 0.001) and delayed lethality until the end (*, *P* = 0.0006). When treated with Δ*cap59*/Δ*sgl1* and Δ*cap59* EVs, animals showed levels of survival similar to those seen with PBS treatment (*P* > 0.01; some animals died between the EV treatment and WT challenge, and the animals that became pupae were excluded from the analysis).

C. neoformans EVs are known to be immunostimulatory components (32). The fact that GXM and SGs are present in EVs (Table 1) led us to analyze if the vesicles could be used as a vaccine instead of the live attenuated mutant cells. Since previous results suggested that CD4^+^ T cell-mediated immunity from the protective effects was not required, we used a model of infection with Galleria mellonella, which lacks T cell-mediated immunity. This model also requires smaller amounts of EVs than the mouse model. Vesicles were isolated from WT, Δ*cap59*/Δ*sgl1*, Δ*cap59*, and Δ*sgl1* cells and used to treat G. mellonella before challenging the animals with WT cells. Vesicle amounts were normalized to the amounts of glucosylceramide (GlcCer) and sterols, which are typical EV components (33). Vesicle fractions for G. mellonella stimulation contained 2 µM sterol, which corresponded to 0.22 pmol of GlcCer (Table 2). G. mellonella survival analysis (Fig. 11) revealed that although all animals died at the end of the experiment, those that received Δ*sgl1* EVs were 100% protected until day 3 (*, *P* = 0.001) and this vaccination resulted in delayed lethality compared to PBS (*, *P* = 0.0006). Interestingly, animals receiving WT EVs died sooner than the ones that received only PBS (*, *P* = 0.0062). Vaccination with Δ*cap59*/Δ*sgl1 and* Δ*cap59* EVs resulted in no protection compared to PBS vaccination (*P* > 0.01).

**TABLE 2:** Quantitative analysis of C19:2/C18:0 GlcCer and ergosterol of purified EVs by MS and Amplex red assay, respectively^*a*^

EV	GlcCer (pmol)	Ergosterol (µM)
Strain WT	1.18	10.91
Strain Δ*cap59*	1.16	7.26
Strain Δ*sgl1*	5.27	76.84
Strain Δ*cap59*/Δ*sgl1*	15.32	89.55

a GlcCer, glucosylceramide.

## DISCUSSION

In this work, we showed that SGs and GXM are functionally related in the Δ*sgl1* mutant. To test whether the accumulation of SGs was itself the only factor that would induce protection in a mouse model, we successfully generated the double mutant Δ*cap59*/Δ*sgl1*. The phenotypic characteristics of the Δ*cap59*/Δ*sgl1* mutant were generally similar to those of the corresponding Δ*cap59* background strain. The Δ*cap59*/Δ*sgl1* mutant was rapidly cleared from the lungs. This response from the host was faster than that seen in the Δ*sgl1* mutant-infected mouse, whereas the Δ*cap59* mutant infection persisted in the lungs throughout the window of observation. Confirming the CFU data, lung histology showed that the *SGL1*-deleted mutants were cleared from the lungs at late stages of the infection, and the immunological characterization showed that the assembled inflammatory responses were clearly different between these mutants. All mutants were nonpathogenic, and, when used as live attenuated vaccines, the Δ*cap59*/Δ*sgl1* mutant showed a loss of protective properties compared to the Δ*sgl1* mutant, which indicates that SGs alone are not sufficient for protection. In fact, GXM seems to play an essential role in protection because when EVs, which notoriously contain GXM (33), were used as vaccine formulations in G. mellonella, we found that EVs enriched in SGs, but lacking GXM, were not able to protect the host in a manner similar to that seen with EVs enriched in both SGs and GXM.

SGs and GXM can modulate the host’s immune system (12, 23). Studies with plant SGs have also suggested a particular role of these immunostimulatory molecules in the balance of Th1 and Th2 immune responses (12, 14–17). Studies in mammalian cells have reported that glycosylation of cholesterol stimulated by Helicobacter pylori may impair phagocytosis, T cell activation, and, ultimately, bacterial clearance (34). However, little has been elucidated about the roles of fungal SGs. Besides being associated with protective effects in a pulmonary cryptococcosis model and with fungal clearance (7), levels of SGs of Pichia pastoris were found to be increased under stress conditions, including heat shock and exposure to ethanol (35). SGs also participate in autophagy-mediated degradation of peroxisomes (36). Accumulation of SGs was observed in a C. neoformans mutant lacking the *APT1* gene, which encodes a putative flippase responsible for the disposition of lipids in the membranes, in an asymmetric way (37). This mutant is hypopathogenic and was associated with reduced GXM production, defective vesicle trafficking, and impaired lipid biosynthesis (37, 38). The lower pathogenicity of the Δ*apt1* mutant associated with increased SG content agrees with our results. Our data also show that accumulation of SGs was evident in cellular and extracellular fractions, including vesicles.

Differences between the Δ*sgl1* and Δ*cap59*/Δ*sgl1* strains in colonizing and in inducing an immune response in our pulmonary cryptococcosis model were also observed. Immune defenses in the lungs at early stages of infection were stimulated in waves, with the aim of maintaining a balance between sufficient protection and minimal damage effects (39, 40). In fact, SGs seem to stimulate proliferation of eosinophils and lymphocyte cells as reported previously (14, 17), but such an increase in cytokine levels (14, 16, 17) was not clearly observed in our model. On the other hand, mice injected with capsular cells showed induction of chemokines. These chemokines are involved in leukocyte recruitment and polarization of Th1/Th2 immune responses to C. neoformans infection, evidencing its central role in cell-mediated immunity (41). However, sustained levels of these cytokines, as observed solely in the WT-infected group, can lead to immunopathology and be associated with the nonresolution of infection and animal death. These results suggest a main role of GXM under conditions of SG accumulation in the early stages of infection. We also suggest that SGs are adjuvants for the protective effects of GXM. The majority of adjuvants activate antigen-presenting cells, mostly dendritic cells, leading to subsequent differentiation of antigen-specific T cells (42, 43). Interestingly, glycolipids have been studied as potential vaccine adjuvants by stimulating the invariant natural killer T (iNKT) cells (42). Several microbial glycolipids are recognized by these cells, which can trigger secretion of Th1 and Th2 cytokines simultaneously, inducing the transactivation of dendritic cells, macrophages, natural killer cells, and B and T cells (42). The effects of a synthetic glycolipid in stimulating iNKT cells revealed an increase in the level of gamma interferon (IFN-γ) and a decrease of in the fungal burden in animals infected with WT C. neoformans cells (44), which suggests that SGs may have been contributing to the clearance of the mutants through this effect.

In our vaccination model, mutant cells lacking *CAP59* were unable to induce protection. These results were different from those obtained by Specht and colleagues (5), who observed partial protection in animals pretreated with the Δ*cap59* strain. This discrepancy might be explained by the usage of different mouse strains (C57BL/6J mice instead of the CBA/J mice used in our study) and the administration route of Δ*cap59* (subcutaneous instead of intranasal in our study).

The mechanism by which GXM is involved in the anticryptococcal protection needs to be further investigated. Early studies have reported the main role of CD4^+^ T cells and Th1 responses in the protection against cryptococcosis in animal models (45–48). We did observe an increase of the CD4^+^ T cell population in the lungs of mice pretreated with the Δ*sgl1* mutant in the vaccination model, which indicates that CD4^+^ T cell are stimulated by GXM-SGs to orchestrate a protective immune response. However, our previous data did show that, under conditions of CD4^+^ T cell depletion, the protection induced by GXM in the presence of the SG accumulation may go beyond the Th1 response, since protection was also previously observed under conditions of CD4^+^ T cell deficiency (7). Interestingly, as observed with the Δ*sgl1* mutant, a C. neoformans strain genetically modified to produce IFN-γ (H99γ) induced protection in CD4^+^ T cell-depleted mice (49). Indeed, CD8^+^ T cells participated in protection in a cryptococcosis model in a manner independent of CD4^+^ T cells but dependent on IFN-γ (50). It is known that CD8^+^ tissue-resident memory T cells (CD8^+^T_RM_) provide local protection against secondary infection (51, 52), and such cells could be involved in the protective effect observed in our study. Different studies have shown that microbial polysaccharides can activate classical and memory CD8^+^ T cells (53, 54); thus, it is possible that C. neoformans GXM could activate lung memory CD8^+^ T cells, particularly when GXM is associated with SGs. In addition, GXM can modulate innate responses through interactions with Toll-like receptors (TLR) 2 and 4 and with CD14, CD18, and FcγRIIB receptors (23, 55, 56). These responses result in the modulation of neutrophils, macrophages, monocytes, and dendritic cells (57, 58). In fact, we did observe massive infiltration of neutrophils and macrophages in the lungs of mice infected with the capsular mutant. The Δ*sgl1* strain induced even greater infiltration than was seen with systems where mice were infected with the WT strain. In association with SGs, GXM bioactivity and binding to immune receptors can be modified, as previously demonstrated after association of the polysaccharide with chitin oligomers (59).

Although GXM-based vaccines seem to protect through antibody-mediated immunity (11, 19, 27), passive transfer of immune serum from animals injected with the Δ*sgl1* strain to naive mice did not induce protection against C. neoformans infection (data not shown), suggesting that the protective effect induced by the Δ*sgl1* strain may not be antibody mediated. However, more studies are necessary to address this issue and to rule out the effect of this mechanism of protection in our model. For instance, analysis of the protective effects observed here in infection models using B cell knockout mice will be crucial to reveal the role of antibodies in SG/GXM-mediated immunological protection against cryptococcosis.

EVs containing SGs and GXM obtained from the Δ*sgl1* mutant delayed lethal infections with C. neoformans in a G. mellonella model. Although the delayed mortality was clear in comparisons between the different protective treatments, all animals ultimately died after challenge with the WT strain. The loss of the protective effect of the administration of EVs in this system may have been due to the lack of adaptive immunity in G. mellonella (60) and/or to insufficient administration of EVs. In fact, only a single injection of EVs was performed. In the mouse vaccination model, the administration of live attenuated Δ*sgl1* cells may continuously produce and release EVs in the airways. The lung fungal burden of Δ*sgl1* cells started to decrease after day 3 (7), suggesting that there is enough time for the production and secretion of EVs containing GXM and enriched in SGs in the airways during this period. Additional studies are under way to verify the protective effect of these enriched EVs on the innate immune cells. In this sense, mice models of EV-mediated protection are likely more informative than the invertebrate model used in our study to reveal the mechanisms of immunological protection. However, a major limitation of murine models of EV-based vaccination is related to the lack of appropriate protocols for obtaining large amounts of EVs. This limitation will be likely overcome in a near future, as a consequence of the recent development of a facilitated protocol of EV isolation from fungal cultures (61).

Curiously, the Δ*sgl1* EV mutants are much larger than the EVs from WT or reconstituted strain (data not shown). This was confirmed *in vitro* when we synthesized an ethanol dilution of small unilamellar vesicles from binary mixtures of purified SGs and GlcCer extracted from C. neoformans according to previously established methods (62). We noted that addition of SGs systematically increased their size and reduced their diffusion coefficient, measured using the Stokes-Einstein equation (63) (see Fig. S5 in the supplemental material). This increase in vesicle size and the observed decrease in diffusion may reduce the speed of vesicle movement and thus increase the duration of interactions between vesicles and host cells. Given the notable immunomodulatory properties of GXM (23, 55) and SGs (32, 64), the increased interaction time might lead to a stronger host immune response, resulting in the elimination of the primary infection and in the stimulation of host protection against a secondary infection.

10.1128/mBio.02909-18.6FIG S5SG addition affected GlcCer vesicle size and diffusion coefficient. (A) Diameter of vesicles synthesized from pure GlcCer and from GlcCer mixed with various amounts of SGs. (B) The diffusion coefficient of the vesicles described in the panel A legend as calculated from the Stokes-Einstein equation ($D=\frac{K_{B}T}{6\pi \mu r}$, where *D* is the diffusion coefficient, *K_B_* is the Boltzmann constant, *T* is the temperature, μ is the viscosity of the blood [3 × 10^−2^ poise for male adults], and *r* is the radius of vesicles). Download FIG S5, PDF file, 0.03 MB.Copyright © 2019 Colombo et al.2019Colombo et al.This content is distributed under the terms of the Creative Commons Attribution 4.0 International license.

EVs from Candida albicans produced protective effects in a G. mellonella model (65). In our study, however, the presence of C. neoformans WT EVs seemed to exacerbate the infection instead of controlling it. In fact, there is experimental evidence that pretreatment with cryptococcal EVs may enhance disease progression in mice (66). Besides, it has been recently reported that the presence of GXM in cryptococcal EVs, but not the presence of GXM alone, augments virulence of C. gattii *in vitro* (67). These functional differences between C. albicans and C. neoformans EVs may be explained by distinct vesicle compositions, including the presence of species-specific proteins and complex carbohydrates, such as GXM (65, 68). Notably, our results showing that EVs enriched in SGs and GXM contributed to disease control suggest that these antigens can also be used in immunotherapeutic approaches against cryptococcosis. However, this hypothesis requires validation in models of antigen administration after challenging mice with C. neoformans.

In conclusion, our study identified GXM as a key factor in induction of protection by a live attenuated C. neoformans vaccine under conditions of SG accumulation. Our results also suggest that the host immune protection most likely initiates in the lung and is perhaps mediated by macrophages, neutrophils, dendritic cells, and CD8^+^T_RM_ but not by CD4^+^ T cells. Further studies are necessary to identify the specific host cellular component(s) responsible for protection. Finally, our studies highlighted the potential use of fungal EVs enriched in GXM and SGs as a potential formulation for fungal vaccines. This cell-free formulation would be particularly attractive in treatment of immunocompromised patients at risk of developing cryptococcosis.

## MATERIALS AND METHODS

### Supplemental materials and methods.

Supplemental materials and methods are described in Text S1 in the supplemental material.

10.1128/mBio.02909-18.1 TEXT S1 Supplemental materials and methods. Download Text S1, DOCX file, 0.02 MB. Copyright © 2019 Colombo et al. 2019 Colombo et al. This content is distributed under the terms of the Creative Commons Attribution 4.0 International license.

### Strains.

The strains used were C. neoformans strain var*. grubii* H99 wild type (WT); mutant Δ*cap59*, an acapsular strain kindly provided by NIH by June Kwon Chung; and mutant Δ*sgl1*, a strain that accumulates sterylglucosides (SGs) and that was developed by our group in Maurizio Del Poeta’s laboratory (7). Growth conditions are detailed in each experimental description.

### Imaging of capsular GXM and purification and analysis of exo-GXM.

**(i) Scanning electron microscopy (SEM).** Fungal cells (10^6^) were cultivated in Sabouraud for 24 h, washed, and transferred to minimal medium (MM), composed of dextrose (15 mM), MgSO_4_ (10 mM), KH_2_PO_4_ (29.4 mM), glycine (13 mM), and thiamine-HCl (3 μM), for 48 h. Cells were washed with PBS and fixed in 0.1 M sodium cacodylate buffer containing 2.5% glutaraldehyde for 1 h. Then, the samples were washed in buffer containing 0.1 M sodium cacodylate, 0.2 M sucrose, and 2 mM MgCl_2_ and fixed on coverslips coated with poly-l-lysine, for 20 min. The preparations were serially dehydrated with alcohol, followed by submission to critical point drying and metallization. A Quanta-FEI scanning electron microscope (FEI, Hillsboro, OR) was used to analyze the cells (69).

**(ii) Exo-GXM purification, quantification, and thin-layer chromatography (TLC).** WT or Δ*sgl1* cells were grown in yeast extract-peptone-dextrose (YPD) overnight and transferred to MM for 48 h. Purification of secreted GXM was done by ultrafiltration as previously described (70). The amount of purified GXM from each strain was measured by phenol-sulfuric acid methodology in 96-well plates as described previously by Masuko and collaborators (71). To characterize the lipids in the purified GXM, 6 mg/ml of GXM from WT and Δ*sgl1* cells was lyophilized and lipid extraction was then performed (see details under “Lipid extraction, mass spectrometry (MS), and TLC” below). The samples were resuspended in 30 μl of CHCl_3_/MeOH (2:1 ratio) and analyzed by TLC, developed with chloroform/methanol/water (65:25:4 [vol/vol/vol]), and stained with iodine and resorcinol.

**(iii) Dynamic light scattering (DLS).** The GXM samples obtained from the different strains were diluted 100-fold in filtered Milli-Q water. The diameter and polydispersity of the obtained polysaccharide suspension were measured by the use of a DynaPro-99 DLS instrument (Wyatt Technology Corporation, Santa Barbara, CA) (72).

### Lipid extraction, mass spectrometry (MS), and TLC.

Lipid extraction was performed before every TLC and MS analysis in this study using the protocols of Singh and colleagues (73). For determination of SG levels in cell pellets, GXM, and EVs, we used different volumes (10 ml, 1 ml, and 50 µl, respectively) from a culture of 1 liter. We dried the samples and extract the lipids for MS analysis. For mutant characterization, we cultivated the strains in yeast nitrogen base (YNB) broth for 24 h at 30°C with shaking and a pellet with 5 × 10^8^ cells was used for lipid extraction. The extraction followed three major steps: Mandala's method (74), followed by the methodology of Bligh and Dyer (75) and then base hydrolysis (73). The samples obtained were resuspended in chloroform/methanol (2:1) for the TLC, and purified SGs and GlcCer were used as references. For MS analysis, a standard lipid mix from Avanti Polar Lipids (Alabaster, AL) was prepared and added to the cells for calibration purposes prior to the first step of lipid extraction. Ergosteryl-3-β-glucosides (SGs) and various sphingolipids and IPC were analyzed as described previously (37, 76). The samples were resuspended in 150 µl of ammonium formate (1 mM)–0.2% formic acid–methanol. The separation was processed through a Thermo Accela high-performance liquid chromatography (HPLC) system (San Jose, CA). The HPLC system was attached to the heated electrospray ionization (HESI) source of a Thermo TSQ Quantum Ultra triple quadrupole mass spectrometer (San Jose, CA). Data were analyzed on Thermo Xcalibur 2.2 Quan Browser software and normalized to inorganic phosphate content (7, 76, 77).

### Purification of EV and quantitative analysis.

EV isolation was performed for the WT, Δ*cap59*, Δ*sgl1*, and Δ*cap59*/Δ*sgl1* strains following the protocol of Rodrigues and collaborators (78). After being maintained for 48 h in YNB, the culture was centrifuged for 15 min at 4,000, 10,000, and 15,000 × *g* to separate the cell debris. The remaining supernatant was concentrated using an Amicon system and a membrane of 100 kDa. Next, the concentrated supernatant was ultracentrifuged at 100,000 × *g* to isolate EVs from the supernatant. Quantification of sterols within the vesicles was performed using an Amplex red kit (Thermo Fisher Scientific), and, for more-specific quantification of the SGs (ergosteryl-3-β-glucosides), MS analysis was used. GlcCer was also analyzed using this technique.

### Deletion of the *SGL1* gene in the Δ*cap59* mutant.

In order to delete the *SGL1* gene (CNAG_05607) in the Δ*cap59* mutant, which is hygromycin resistant, we used the same disruption cassette, p*sgl1* (pCR-5UTR-NAT1-3UTR-TOPO), and a protocol similar to that used in the previously reported work involving the Δ*sgl1* mutant (7). Briefly, we used a biolistic DNA delivery method (79) to introduce the plasmid psgl1 into the Δ*cap59* mutant. Transformants from different colonies were cultivated in YPD and NAT-containing YPD plates (100 μg/ml) to check stability and to select the colonies of transformants. The genomic DNA of these selected colonies was then isolated for Southern blot analysis. We used EcoRV and KpnI restriction enzymes to digest the DNA and two different probes to check the correct insertion of the fragment. We identified a band in the Southern blot using a 5′ untranslated region (5′UTR) probe of 4,910 bp for the fungal cells that had the *SGL1* gene intact and without any alteration in the gene sequence and a band of 3,604 bp for the fungal cells with a deletion of *SGL1* and an insertion of NAT cassette in the correct position. For the gene probe, the cells that had the *SGL1* gene disrupted did not present any visual band.

### Double mutant characterization.

**(i) Growth curve.** Fungal cells were grown in YPD overnight at 30°C with shaking. They were washed with PBS, and 10^5^ cells/ml were added into 10 ml of Dulbecco's modified eagle medium (DMEM) at pH 4 or pH 7.4 at 37°C and 5% CO_2_. Samples of 100 µl were taken after 24, 48, or 72 h and plated in YPD plates for CFU verification.

**(ii) Morphology and capsule analysis.** The cells were growth in YPD overnight at 30°C with shaking or in DMEM media at pH 4 or pH 7.4 at 37°C and in the presence of 5% CO_2_. The capsule was analyzed through counterstaining of the cells with India ink and examination under ×100 magnification. In addition, transmission electron microscopy (TEM) was performed to further characterize the capsule and cell structure. The protocol for the agarose embedding was similar to that described by Munshi and colleagues (76), and pictures were taken at magnifications of ×23,000 and ×49,000. A Leica EM UC7 ultramicrotome was used for sectioning. The sections were placed on 300-mesh copper grids and stained with uranyl acetate and lead citrate.

**(iii) Characterization of virulence factors.** Melanin and urease assays were performed at 37°C with 5% CO_2_. Plates for melanin testing were prepared with MM agar with addition of 1 mM l-3,4-dihydroxyphenylalanine (l-DOPA). Fungal cells were spotted at different dilutions (10^7^, 10^6^, 10^5^, 10^4^, and 10^3^) and visually analyzed after 96 h of cultivation. The production of melanin was determined by the dark color of the cells (80). Plates for urease test were prepared with Christensen's urea agar, and 5 × 10^4^ cells were spotted in the center of the plate. Cells were cultivated for 120 h and assessed by difference represented by the pink color of the colony and the size of the pink halo (81, 82).

### Mice.

CBA/J mice (Envigo, Indianapolis, IN) (3 to 4 weeks of age) were used in this study. The animals had access to food and water *ad libitum*. All animal procedures were approved by the Stony Brook University Institutional Animal Care and Use Committee (protocol no. 341588) and followed the guidelines of American Veterinary Medical Association.

### Infection, fungal burden, and histology.

All the infections were performed intranasally. Animals were anesthetized with 60 µl xylazine/ketamine solution intraperitoneally (95 mg of ketamine and 5 mg of xylazine per kilogram of animal body weight). Fungal cells, including WT, Δ*cap59*, Δ*sgl1*, and Δ*cap59*/Δ*sgl1* cells, were grown overnight in YPD, counted, and resuspended in PBS. Inoculation of 7 × 10^5^ cells per animal in a volume of 20 µl was performed through nasal inhalation. Animals were checked daily, and those that had more than 20% weight loss and appeared moribund or in pain were euthanized using CO_2_ inhalation followed by cervical dislocation. For survival curve analysis, 8 animals per group were monitored during 60 days after infection. After this period, the lungs and brains of the animals that had survived (2 per group) were used for histology analysis. Hematoxylin and eosin (H&E) and periodic acid–Schiff (PAS) staining was performed. Histological analyses of the lung and brain of animals injected with the strains were also performed after 3 days of infection. Because the WT-injected mice were getting sick and dying at around day 14, we also performed histology in this time point for this strain. For fungal burden studies, 3 animals per strain were used at each time point. The homogenization of lung and brain was performed with 10 ml of PBS using a Stomacher 80 blender (Seward, United Kingdom). The samples were plated on YPD plates for CFU analysis after 48 h.

### Vaccination in murine model.

For vaccination studies, we infected 10 mice with PBS or with Δ*cap59*, Δ*sgl1*, and Δ*cap59*/Δ*sgl1* cells intranasally. We used the same techniques and numbers of cells used for survival curve analysis. After 30 days, animals were rechallenged with 7 × 10^5^ cells of the WT strain. Survival of mice was monitored for 60 days after the rechallenge.

### Lung immune cell populations and cytokine analyses.

The detection of immune cell populations and cytokines was performed after pulmonary infection model and after WT rechallenge in the vaccination model. The procedures used for the two models were the same as described previously, except for the inoculation of 5 × 10^5^ fungal cells and the time points. Separate groups of 3 mice per treatment each day were used for the two analyses. For the last time point (30 days), we did not have the WT-treated group since those mice had succumbed to infection previously. In the vaccination model, at 30 days after the WT challenge, only the Δ*sgl1*-pretreated mice were alive. The immune cell populations were analyzed by flow cytometry. Lungs were harvested into sterile PBS, minced in fluorescence-activated cell sorter (FACS) buffer with added collagenase and DNase I, homogenized through a 70-μm-pore-size filter, lysed in Ack lysis buffer, washed with PBS, and filtered again, leaving a single-cell suspension. The cell suspensions were subjected to Fc blocking and then stained with a fluorescence-labeled antibody cocktail. The antibody cocktail included viability stain AF700, CD103-allophycocyanin (CD103-APC), major histocompatibility complex class II-fluorescein isothiocyanate (MHCII-FITC), LY6C-peridinin chlorophyll protein-Cy5.5 (LY6C-PerCPCy5.5), Ly6G-phycoerythrin (Ly6G-PE), Siglec F-PE Texas Red, CD11c-PE Cy7, F4/80-BV421, CD11b-BV510, CD4-BV605, CD19-BV650, CD45-BV711, and CD8-BV785. The stained cells were resuspended in FACS buffer, run on a BD LSR II flow cytometer (BD Biosciences), and analyzed using Flowjo version 10. The detection of cytokines in the lung tissue was analyzed using a Milliplex Mouse Cytokine/Chemokine Magnetic Bead Premixed 25 Plex kit (Millipore Sigma), following the 2-h incubation protocol and using PBS as the matrix for the samples. The samples were immediately run on a Bioplex 200 system running Bioplex manager version 4.1.1. Before homogenization of the lungs, BAL fluid was collected for a separate analysis (see the figures in the supplemental material). Cytokine measurements that fell either above or below the detection limits were excluded to reduce bias; therefore, the graphical illustrations of all relevant cytokines portray values within the detection limits of the assay.

### Vaccination study with the EVs in an invertebrate model.

For vaccination study with the EVs, G. mellonella was used in its final instar larval stage (60). Thirty larvae were injected with 10 μl of PBS or an EV suspension of WT, Δ*cap59*, Δ*sgl1*, or Δ*cap59*/Δ*sgl1* containing 2 μM sterol (based on WT quantification). Injection into the hemocoel through the prolegs was performed using a Hamilton syringe. The animals were kept in a 37°C incubator in the dark. After 24 h, larvae were injected with 10 μl of PBS containing 2 × 10^4^ cells of the WT strain. The larvae were monitored daily for survival.

### Statistical analysis.

Statistical analysis was performed using GraphPad Prism software version 7.0. Data are presented as means ± standard errors of the means. One-way analysis of variance (ANOVA) or a two-way repeated-measure ANOVA was performed followed by a Tukey’s *post hoc* test for multiple comparisons. For survival curve determinations, statistical analysis was performed using the log rank (Mantel-Cox) test for survival analyses, and data represent the mean percentages of survival.
