# Supplementary figures and images for: Cryptococcus neoformans Glucuronoxylomannan and Sterylglucoside Are Required for Host Protection in an Animal Vaccination Model
Source: mBio. 2019 Apr 2;10(2):e02909-18. doi: 10.1128/mBio.02909-18 (PMC6445945; doi:10.1128/mBio.02909-18)

**A.**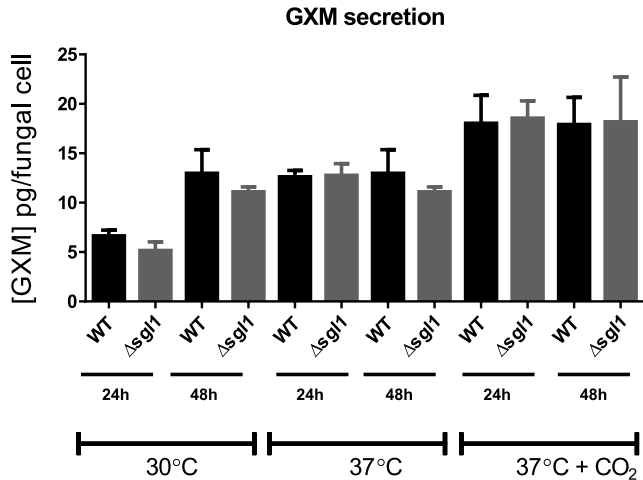**B.**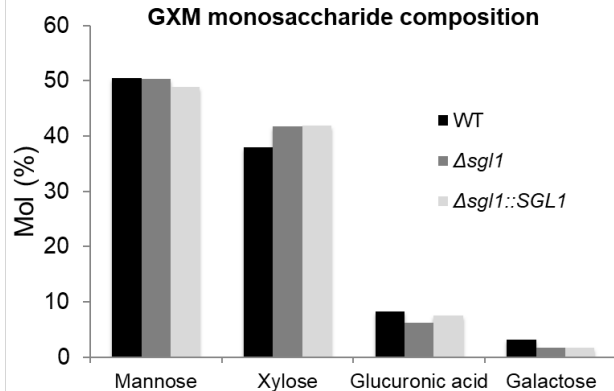

Supplement: FIG S1 [file mBio.02909-18-sf001.pdf]

**A.**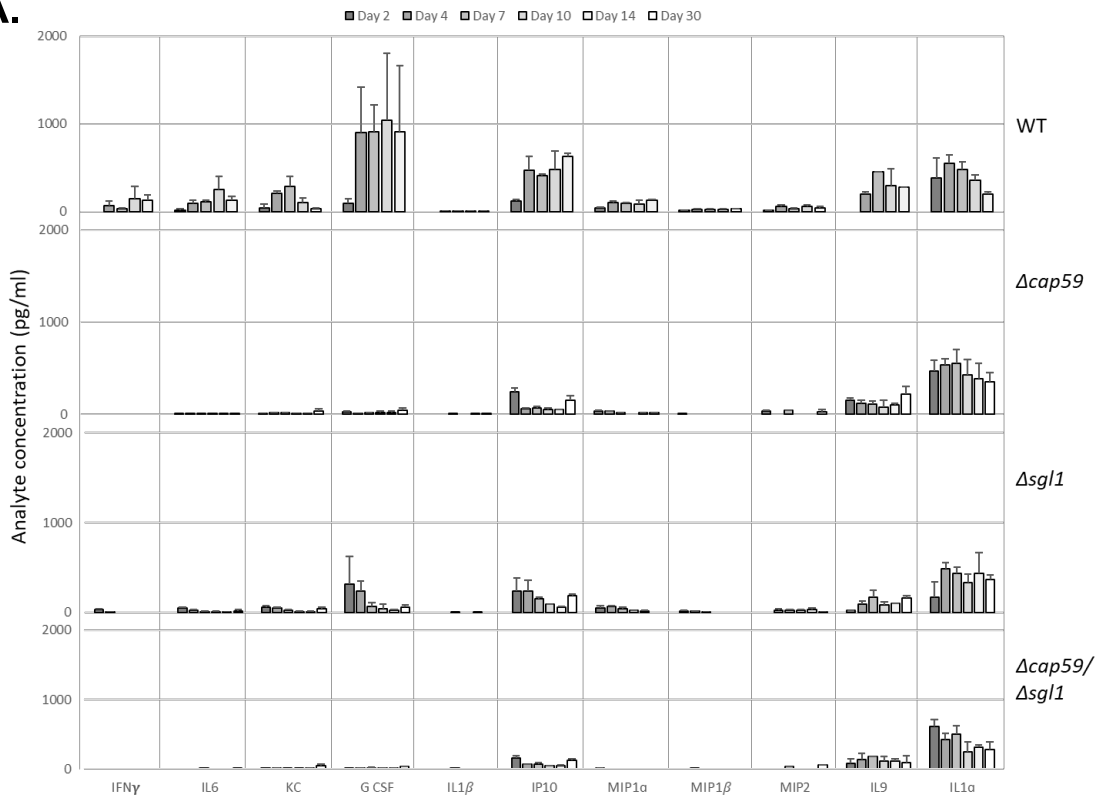**B.**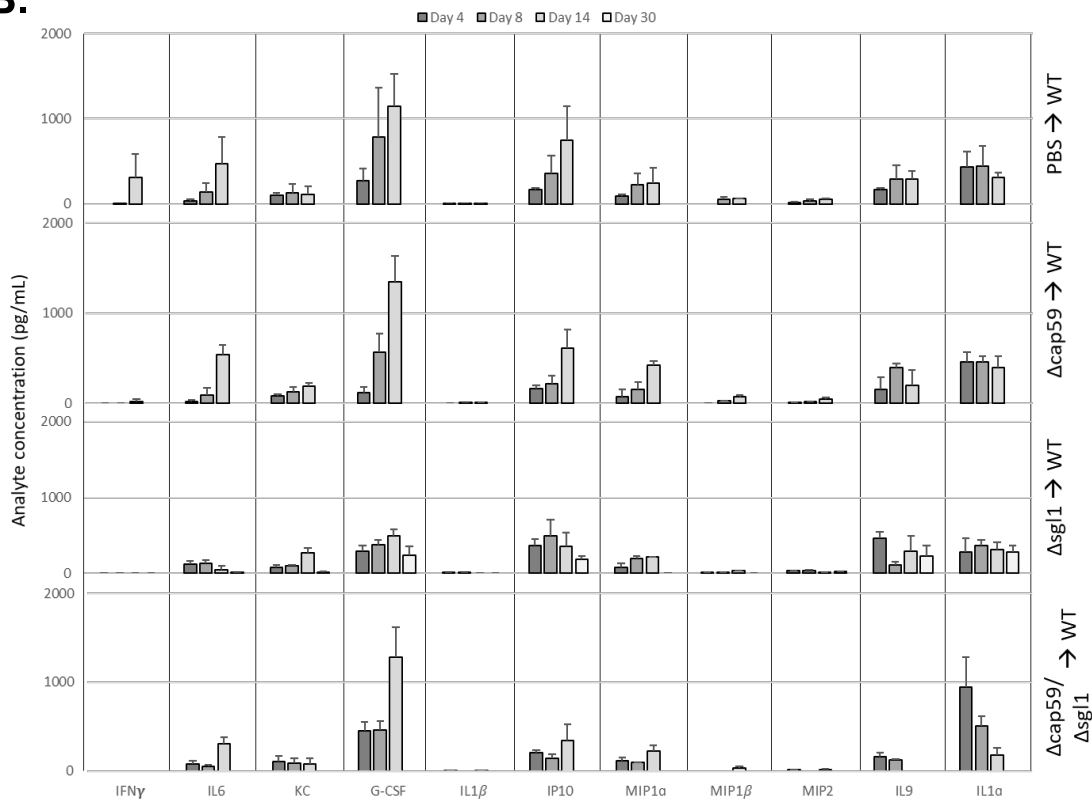

Supplement: FIG S2 [file mBio.02909-18-sf002.pdf]

**A.**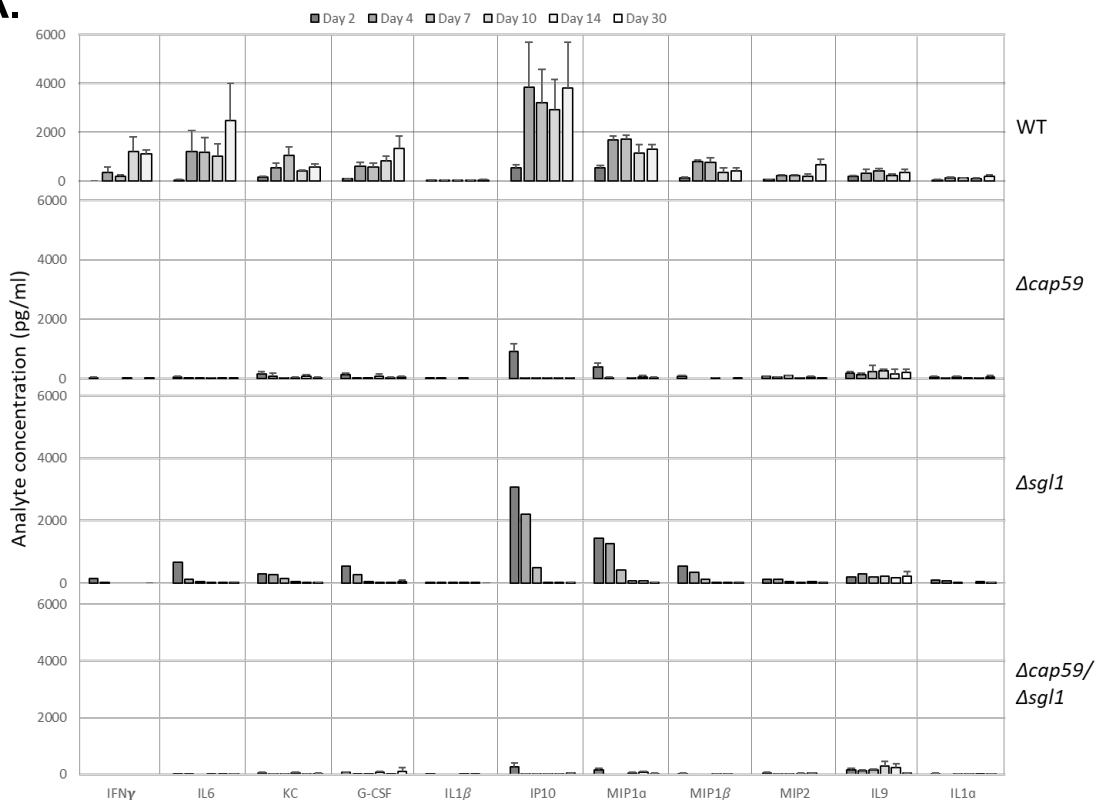**B.**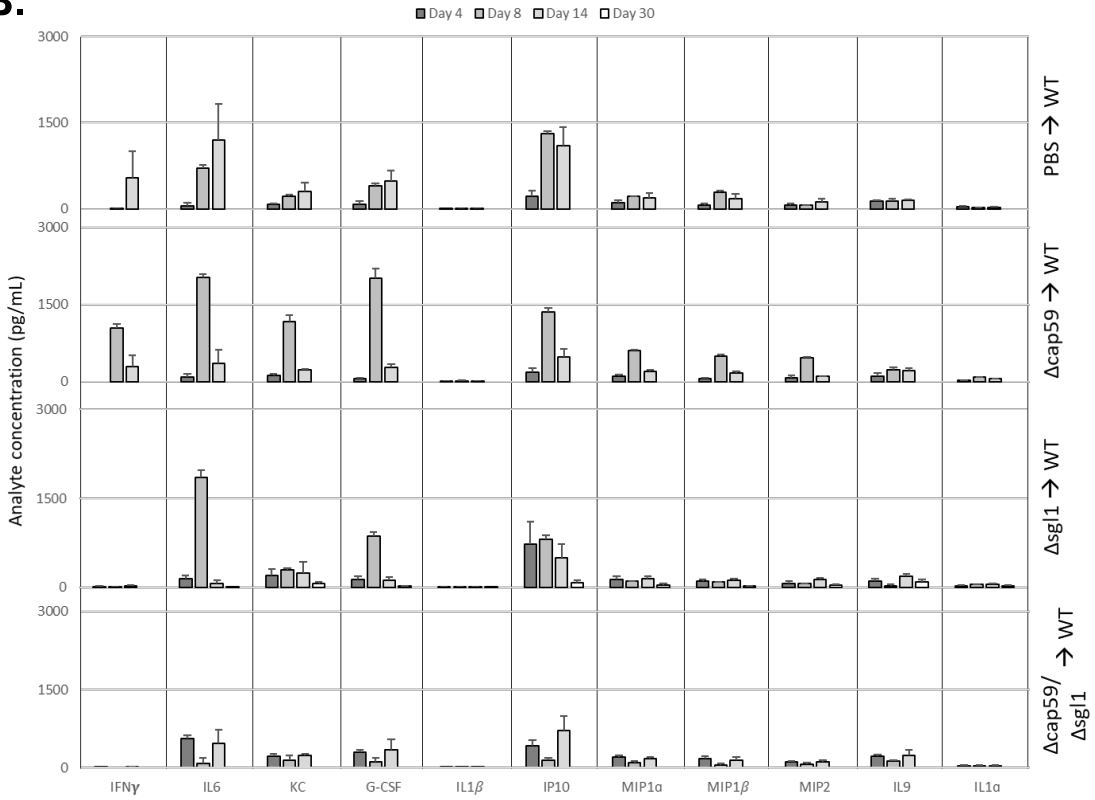

Supplement: FIG S3 [file mBio.02909-18-sf003.pdf]

**A.**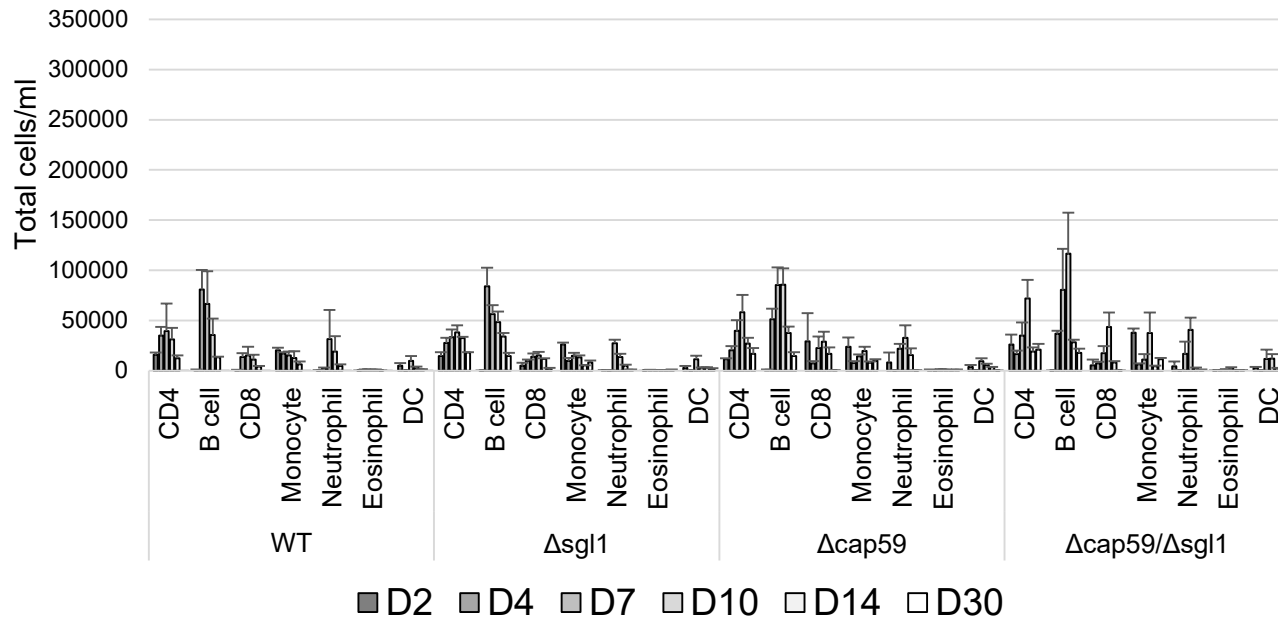**B.**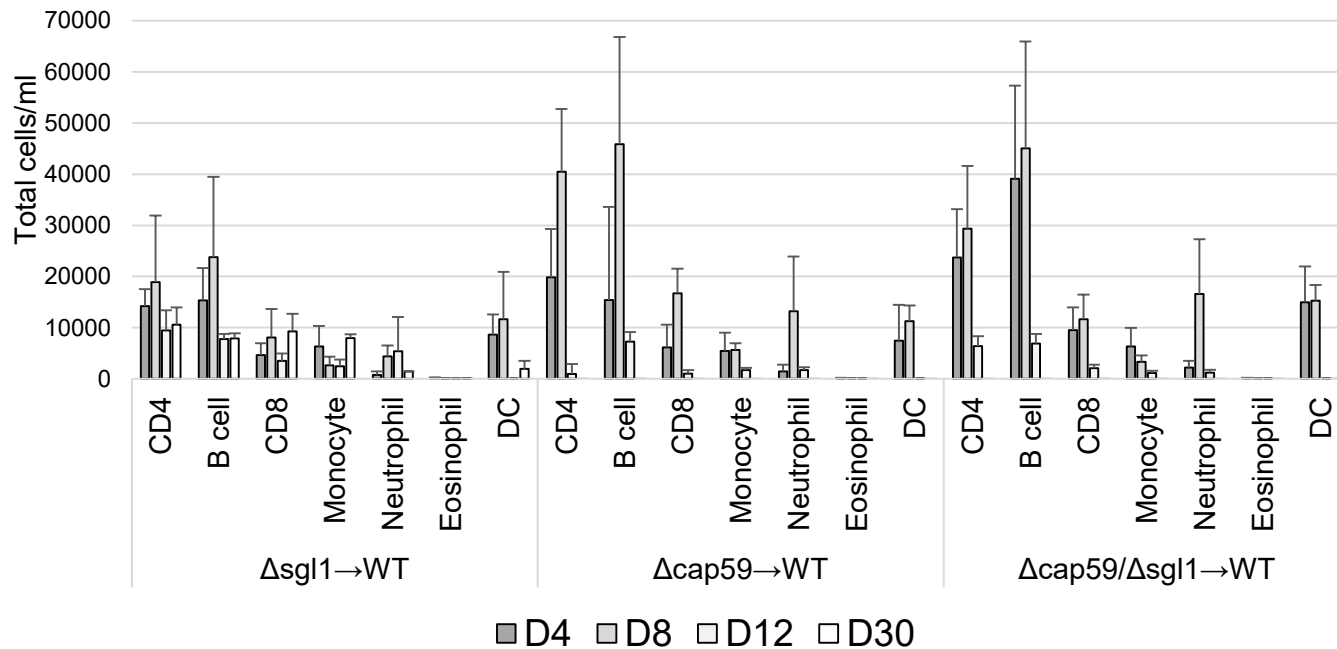

Supplement: FIG S4 [file mBio.02909-18-sf004.pdf]

**A.**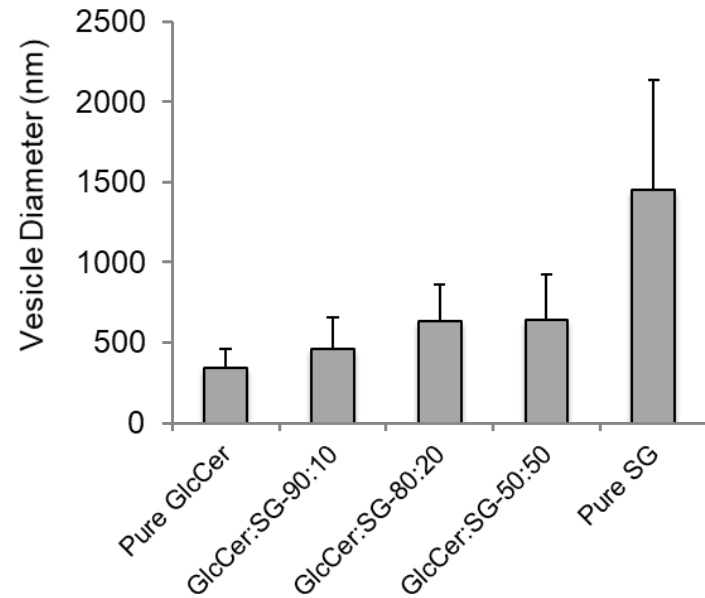**B.**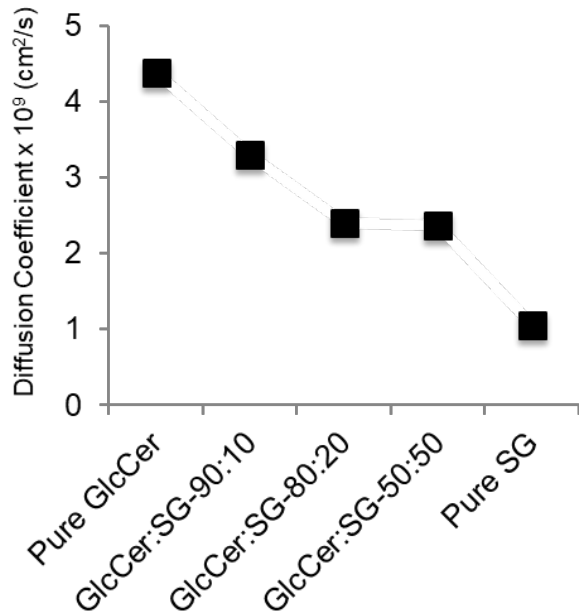

Supplement: FIG S5 [file mBio.02909-18-sf005.pdf]
