# Supplementary material for: Cryptococcus neoformans Glucuronoxylomannan and Sterylglucoside Are Required for Host Protection in an Animal Vaccination Model
Source: mBio. 2019 Apr 2;10(2):e02909-18. doi: 10.1128/mBio.02909-18 (PMC6445945; doi:10.1128/mBio.02909-18)
Supplement: TEXT S1 [file mBio.02909-18-s0001.docx]

SUPPLEMENTAL MATERIAL

MATERIALS AND METHODS

**GXM secretion and composition and supernatant viscosity.**

*ELISA for GXM secretion.* WT or Δsgl1 were grown in YPD followed by MM for 24 h and 48 h at 30°C, 37°C, and 37°C + CO_2_. Aliquots of cultures supernatant were analyzed for GXM quantification by ELISA. The cultures supernatants after 24 h growth were diluted 1:50 and those of 48 h were diluted 1:100 in a 96-well plate and incubated for 1 h at 37^o^C. The wells were blocked with PBS-BSA 1% for 1 h at 37^o^C and 1 μg/ml of mAb 18B7 (in PBS-BSA 1%) was added onto the plate for 1 h at 37^o^C. The plate was washed with PBS-Tween 20 3 times and 50 μl of anti-mouse-HRP (1:5,000 dilution in PBS-BSA 1%) were added to the wells. After incubation at 37^o^C for 1 h the plate was washed with PBS-T, as described above, and 50 μl of [3,3′,5,5′-tetramethylbenzidine](http://www.sigmaaldrich.com/catalog/product/sigma/t0440?lang=en&region=US) (TMB) were added as a substrate to the enzyme horseradish peroxidase (HRP). The reactions were stopped by adding 50 μl of sulfuric acid (0.16M) to each well and the plate was read at 450 nm.

*Gas chromatography-mass spectrometry (GC-MS) analysis.* The monosaccharide composition of purified exo-GXM fraction from WT, Δ*sgl1,* and Δ*sgl1*::SGL1 strains was determined by GC-MS, following methanolysis and derivatization with trimethylsilane (TMS) (M. A. Ferguson, S. W. Homans, R. A. Dwek, T. W. Rademacher, Science, 239:753-759, 1988, ISSN: 0036-8075; M. M. Medeiros, J. R. Peixoto, A. C. Oliveira, L. Cardilo-Reis, V. L. Koatz, L. Van Kaer, J. O. Previato, L. Mendonca-Previato, A. Nobrega and M. Bellio, J Leukoc Biol, 82:488-496, 2007, doi: 10.1189/jlb.0706478). Details of the analysis was described on previous work (L. Nimrichter, S. Frases, L. P. Cinelli, N. B. Viana, A. Nakouzi, L. R. Travassos, A. Casadevall and M. L. Rodrigues, Eukaryot Cell, 6:1400-1410, 2007, doi: 10.1128/EC.00122-07).

*Dynamic viscosity.* The Δ*sgl1* and Δ*sgl1+SGL1* strains were grown in YPD for 48 h at 30^o^C. Purification of secreted GXM was performed as described before. We used the concentrate supernatant obtained after use of an Amicon system and a membrane of 100 kDa. Around 200 μl of supernatant of each mutant was mixed with 10 μl of trypan blue, and 30 µl of this mixture was applied on an inclined glass surface at the same time, for viscosity comparison and video recording.

**Blood immune cell populations and cytokine analysis.** The same experimental design was performed as described for the lung analysis. For flow cytometry, the blood was collected via a cardiac puncture and stored in heparin-coated tubes on ice. For each sample, 100 μl of blood was centrifuged at 1000 rcf for 10 mins at 4°C to separate the serum fraction. The red blood cell fraction was collected, lysed in Ack lysis buffer [8.29g/L NH_4_Cl, 1g/L KHCO_3_, 37.2mg/L EDTA, pH 7.4], washed in sterile PBS, and filtered through 70 μm filter leaving a single-cell suspension. After cell suspension, blockage, staining, and analysis were similar to the lung described previously. For cytokine detection, the same Milliplex Mouse Cytokine/Chemokine Magnetic Bead Premixed 25 Plex kit (Millipore Sigma) was used. Blood serum was collected following the manufacturer’s protocol. In short, blood was collected via cheek bleeding mice with a 4 mm lancet into polypropylene tubes, letting the blood sit at room temperature for 20 mins (allowed blood clotting), and centrifuged at 1500 rcf for 10 mins at 4°C. The serum was collected and stored at -80°C until it was analyzed.

**Vesicle size and diffusion coefficient.** Multilamellar vesicles (MLVs) were prepared from purified glucosylceramide, sterylglucoside, or a mixture of the two lipids. Lipids were purified according to the methods described previously (S. Raj, S. Nazemidashtarjandi, J. Kim, L. Joffe, X. Zhang, A. Singh, V. Mor, D. Desmarini, J. Djordjevic, D. P. Raleigh, M. L. Rodrigues, E. London, M. Del Poeta and A. M. Farnoud, Biochim Biophys Acta, 1859: 2224-2233, 2017, doi: 10.1016/j.bbamem.2017.08.017; A. Rella, V. Mor, A. M. Farnoud, A. Singh, A. A. Shamseddine, E. Ivanova, N. Carpino, M. T. Montagna, C. Luberto, M. Del Poeta, Front Microbiol, 6:836, 2015, doi: 10.3389/fmicb.2015.00836). MLVs were synthesized according to the methods of P. Pathak and E. London (Biophys J, 101: 2417-2425, 2011, doi: 10.1016/j.bpj.2011.08.059). Briefly, lipids (100 μM) were pipetted into glass tubes and dried under nitrogen and then further dried under high vacuum for 2 h. Lipids films were suspended in 70°C phosphate-buffered saline (PBS, pH 7.4) and were allowed to cool down to room temperature prior to measuring their size using dynamic light scattering. Using the size values, the diffusion coefficient of vesicles in blood was estimated using the Stokes-Einstein equation ($D=\frac{K_{B}T}{6\pi\mu r}$), where D is the diffusion coefficient, K_B_ is the Boltzmann constant, T is the temperature, $\mu$ is the viscosity of the blood (3x10^-2^ poise for male adults) and r is the radius of vesicles.
